# Stress Reduction in Perioperative Care: Feasibility Randomized Controlled Trial

**DOI:** 10.2196/54049

**Published:** 2025-01-07

**Authors:** Haridimos Kondylakis, Irene Alice Chicchi Giglioli, Dimitrios Katehakis, Hatice Aldemir, Paul Zikas, George Papagiannakis, Santiago Hors-Fraile, Pedro L González-Sanz, Konstantinos Apostolakis, Constantine Stephanidis, Francisco J Núñez-Benjumea, Rosa M Baños-Rivera, Luis Fernandez-Luque, Angelina Kouroubali

**Affiliations:** 1 FORTH-ICS Heraklion Greece; 2 Adhera Health Inc Santa Cruz, CA United States; 3 ORamaVR SA Heraklion Greece; 4 Innovation & Data Analysis Unit Institute of Biomedicine of Seville IBiS/Virgen Macarena University Hospital/CSIC/University of Seville Seville Spain; 5 Polibienestar Institute University of Valencia Valencia Spain

**Keywords:** CARINAE, digital health, patient empowerment, stress and anxiety management, mHealth, virtual reality, feasibility, perioperative, randomized controlled trial, surgery, risk, patient empowerment, wearable devices, patient education, mobile app, psychological, self-efficacy, self-management, well-being, patient monitoring

## Abstract

**Background:**

Patients undergoing surgery often experience stress and anxiety, which can increase complications and hinder recovery. Effective management of these psychological factors is key to improving outcomes. Preoperative anxiety is inversely correlated with the amount of information patients receive, but accessible, personalized support remains limited, especially in preoperative settings. Face-to-face education is often impractical due to resource constraints. Digital health (DH) interventions offer a promising alternative, enhancing patient engagement and empowerment. However, most current tools focus on providing information, overlooking the importance of personalization and psychological support.

**Objective:**

This study aimed to assess the viability of a DH intervention known as the Adhera CARINAE DH Program. This program is specifically designed to offer evidence-based and personalized stress- and anxiety-management techniques. It achieves this by using a comprehensive digital ecosystem that incorporates wearable devices, mobile apps, and virtual reality technologies. The intervention program also makes use of advanced data-driven techniques to deliver tailored patient education and lifestyle support.

**Methods:**

A total of 74 patients scheduled for surgery across 4 hospitals in 3 European countries were enrolled in this study from September 2021 to March 2022. Surgeries included cardiopulmonary and coronary artery bypass surgeries, cardiac valve replacements, prostate or bladder cancer surgeries, hip and knee replacements, maxillofacial surgery, and scoliosis procedures. After assessment for eligibility, participants were randomized into 2 groups: the intervention group (n=23) received the Adhera CARINAE DH intervention in addition to standard care, while the control group (n=27) received standard care alone. Psychological metrics such as self-efficacy, self-management, and mental well-being were assessed before and after the intervention, alongside physiological markers of stress.

**Results:**

The intervention group demonstrated significant improvements across several psychological outcomes. For example, Visual Analogue Scale Stress at the hospital improved at admission by 5% and at hospital discharge by 11.1% and Visual Analogue Scale Pain at admission improved by 31.2%. In addition, Hospital Anxiety and Depression Scale Anxiety after surgery improved by 15.6%, and Positive and Negative Affect Scale-Negative at hospital admission improved by 17.5%. Overall, patients in the intervention study spent 17.12% less days in the hospital. Besides these individual scores, the intervention group shows more positive relationships among the psychological dimensions of self-efficacy, self-management, and mental well-being, suggesting that the CARINAE solution could have a positive effect and impact on the reduction of stress and negative emotions.

**Conclusions:**

Our results provide an important first step toward a deeper understanding of optimizing DH solutions to support patients undergoing surgery and for potential applications in remote patient monitoring and communication.

**Trial Registration:**

ClinicalTrials.gov NCT05184725; https://clinicaltrials.gov/study/NCT05184725

**International Registered Report Identifier (IRRID):**

RR2-10.2196/38536

## Introduction

Patients undergoing surgical operations commonly experience symptoms of severe stress, anxiety, and fear due to the potentially threatening nature of surgeries [1,2]. Family caregivers, who play a crucial role in caring for these patients, also face emotional distress and physical challenges [3]. Surgeons and health care professionals use various stress coping strategies, as stressors can affect surgical performance and lead to complications [4]. Psychological support and patient education have proven effective in reducing stress and anxiety in surgical settings [5,6]. Providing information about the surgical procedure is essential for both patients and caregivers, as it helps decrease anxiety levels and surgical complications [7,8]. Patient empowerment, where patients actively participate in managing their diseases, leads to better self-care management and improved outcomes in terms of satisfaction, cost, health status, and function [9,10]. Addressing caregiver strain is also vital for pediatric patients [11] and those with special health care needs [12].

Being in unfamiliar surroundings and facing preoperative requirements can add to feelings of confusion and stress. It is common for individuals anticipating surgery to experience uncertainty, fear, hesitation, and anxiety. These emotions can affect patients’ well-being and their ability to adhere to instructions throughout their perioperative journey. Research shows [13] that patient experience is closely tied to emotional health and surgical outcomes. Failure to address anxiety and stress adequately can lead to unnecessary discomfort, prolonged hospital stays, higher costs, and suboptimal clinical results.

Preventing presurgical anxiety can significantly contribute to positive outcomes in terms of health and well-being. Various strategies and techniques have been used to manage preoperative stress and anxiety, ranging from simple methods like listening to music and basic relaxation techniques to more involved approaches such as providing information, consulting with nurses, and using advanced interventions like patient education programs and modern information and communication technology tools [14,15].

For instance, allowing preoperative patients the opportunity to listen to music before surgery can prove to be an effective intervention in reducing anxiety and aiding patients in coping with what could potentially be a challenging or stressful procedure. Music has demonstrated its ability to alleviate anxiety among preoperative patients on numerous occasions [8]. However, it is noteworthy that only a small fraction of patients receive adequate stress relief support before undergoing surgery.

Digital health (DH) interventions have significantly supported enhancing health condition awareness and mental health management, using both nonimmersive systems, for example, mobile apps, and immersive systems, for example, virtual reality (VR) [16]. These interventions have shown promise in supporting patients in managing anxiety, stress, and pain [17-19]. Especially for VR, studies [20-23] have demonstrated benefits in various health care areas, including stress and pain reduction [23,24], medical practitioner training, patient counseling, cognitive rehabilitation, physical therapy, dentistry, mental health management, and surgery, as well as in managing pain and anxiety in pediatric patients [21,22,25]. VR’s distractive properties make it an efficient tool for stress relief and pain management [26]. In addition, VR has been shown to reduce perceptions of anxiety in preoperative patients [27].

On the other hand, DH interventions generate vast amounts of data that can be used for personalization through artificial intelligence techniques [28-33]. These techniques capitalize on remote activity recognition and monitoring in order to provide recommendations, enabling the personalization of patient interventions based on their unique behavioral and health needs [34,35]. Perioperative stress varies greatly among patients, depending on the severity of their illness and the type of surgery required. Personalized interventions targeting stress management should take these individual differences into account, incorporating health behavior theories. Health Recommender Systems, like the I-Change model, guide the personalization of educational and behavioral interventions [36,37].

Independent of the progress in the domain, the feasibility of combining VR with mobile-based technologies as the primary means of delivering a DH intervention remains poorly understood [38]. This study aims to explore the feasibility of a DH intervention named CARINAE, which combines evidence-based perioperative stress management, anxiety, and pain relief techniques grounded in behavioral science. The intervention leverages mobile and VR technologies to help patients manage stress and anxiety during surgery, promoting healthy recovery, extensively reported already in another paper [39]. Through self-reported measurements and a control group, the study assesses the impact of the CARINAE platform on perioperative stress, aiming to determine the effectiveness of the intervention.

## Methods

### Overview

In this section, we summarize the design and the protocol followed for the intervention. For more information on the details of the protocol and the technological solution, the interested reader is forwarded to the relevant paper [39].

### Ethical Considerations

This study was approved by the Institutional Review Board of the four hospitals participating in the study (Hospital Universitario Reina Sofia, Spain; Instituto di Ricovero e Cura per Anziani, Italy; Sant Joan de Déu Hospital, Spain; and Fundació Parc Taulí, Spain) and with the 1964 Declaration of Helsinki and its later amendments or comparable ethical standards. Informed consent was obtained from all individual participants included in the study. Additionally, measures were taken to ensure the confidentiality and anonymity of patient data throughout the study. The study posed no significant risks to participants, and they were free to withdraw at any time without affecting their standard of care. Finally, no financial compensation was provided to the participants of the study.

### Study Setting

The study, following ethical boards’ approval, was conducted at the following European hospitals: (1) Hospital Universitario Reina Sofia (SAS; Spain)—Cardiothoracic Surgery Department; (2) Instituto di Ricovero e Cura per Anziani (INRCA; Italy)—Urology Department; (3) Sant Joan de Déu Hospital (SJD; Spain)—Orthopedics and Traumatology Department for Children; and (4) Fundació Parc Taulí (Parc Taulí; Spain)—Orthopedics and Traumatology Department for Adults. A fifth hospital had granted ethical approval to conduct the study; however, due to external reasons, no patients were recruited from this hospital, and so it did not participate.

The recruitment process started in September 2021 and finished in March 2022.

Clinical investigators prescreened the eligibility of the participants that they had available in their pool of participants that were proposed for one of the surgeries in the inclusion criteria that are reported in the sequel. Whenever a potentially eligible participant was identified, he or she was invited to the study, either by phone or during the routine consultation, whatever was more convenient. As soon as the patient showed interest in taking part in the study, she or he was referred to the research coordinator of the study who facilitated him or her by providing the patient information letter and the informed consent form and who solved any questions and concerns that the patient might have. Upon the signature of the informed consent, the patient was considered recruited for the trial.

### Study Design

The CARINAE DH platform was tested in a multicentric trial conducted in 4 clinical settings across two European countries. The trial used a stratified randomized controlled design and aimed to address two main research questions.

RQ1: The first question seeks to determine the extent to which CARINAE impacts patients’ stress, anxiety, and pain levels compared to those who receive the standard of care only. Additionally, the study investigated the effects on secondary outcomes, such as well-being and overall quality of life. As a side effect, the study investigated the impact of CARINAE on caregivers’ stress and anxiety based on the groups they were assigned to.

RQ2: The second research question focuses on evaluating the overall usability of the CARINAE solution based on feedback and experiences from patients, caregivers, and health care professionals involved in the trial.

### Eligibility Criteria

#### Inclusion Criteria

Participants aged 12 to 65 years who underwent various surgeries, including cardiopulmonary bypass surgery, coronary artery bypass surgery, cardiac valve replacement, prostate, kidney, or bladder cancer surgery, hip or knee replacement, maxillofacial surgery, orthognathic surgery, or scoliosis. Adult participants were required to have an Android smartphone and demonstrate basic digital literacy, while children’s caregivers also needed to have an Android smartphone and basic digital literacy.

#### Exclusion Criteria

Participants who could not provide informed consent, communicate effectively in the native language, demonstrate basic digital literacy, exhibited symptoms of dementia, had allergies to dedicated wearable materials like steel and silicone, were pregnant, or were already enrolled in another clinical trial.

### Interventions

#### Overview

The eligible participants were randomly allocated to either the experimental group or the control group using block randomization with a block size of 4. The randomization process was facilitated through the Sealed Envelope web-based tool [31]. The DH solution consisted of three distinct components: a mobile app, a VR component, and a clinical web application. Participants in the experimental group used the first and second components, while the third component was exclusively used by health care professionals for those patients. Both the control and experimental groups underwent the same visit schedule.

#### Control Group

The control group received standard care, which included four visits with the health care provider. During these visits, patients in the control group received instructions on diet and healthy lifestyle habits. In current health care settings, it is not common to provide patients with stress and anxiety relief support during the perioperative period. Assessments for the control group took place during the following visits: (1) the initial visit, where the health care provider communicated the need for surgery to the patient (2-4 weeks before the surgery); (2) hospital admission, which occurred 1-3 days before the surgery; (3) hospital discharge, approximately 1 week after the surgery; and (4) remote follow-up 14 days after the surgery. After each visit, patients were administered several questionnaires capturing primary and secondary outcomes, as well as covariates.

#### Experimental (Intervention) Group

The participants in the experimental group received the digital solution CARINAE during the first visit, along with training on how to use the tool effectively. They were allowed to take CARINAE home and use it as frequently as desired. Following each of the 4 standard care visits, the experimental group completed the same questionnaires as the control group.

#### Sample Size Calculation

The study was a feasibility clinical trial, with the number of participants established before the beginning of the project by the clinics. According to the study protocol reported by Kondylakis et al [39], participants were randomized to achieve a balance between the 2 groups (intervention and control groups) according to the type of surgery and baseline characteristics (covariates). Considering a 1:1 random allocation, significance level of .05 (2-sided), and 80% power, 60 participants were needed to detect a 10% difference in stress, anxiety, and pain between the intervention and control groups. Sample size calculations are estimated using G*Power (version 3.1.9.2; University of Dusseldorf).

### Outcome Variables

The primary outcome variables in this study were stress, anxiety, and pain, which were measured using paper-and-pencil questionnaires. The secondary outcome variables included overall quality of life, emotional status, mental well-being, self-efficacy perception, and patient activation during and after the hospital stay.

For assessing the primary outcome variables, the following questionnaires were used after each standard care visit and administered on paper:

- Patients’ and caregivers’ self-reported stress measured using a Visual Analogue Scale (VAS) at baseline, admission for surgery, hospital discharge, and 2 weeks after surgery [40].
- Patients’ self-reported pain measured using a VAS at baseline, admission for surgery, hospital discharge, and 2 weeks after surgery [41].
- Patients’ Hospital Anxiety and Depression (HADS) measured at admission for surgery, hospital discharge, and 2 weeks after surgery [42].

For assessing the secondary outcome variables, the following questionnaires were used after each standard care visit and administered on paper:

- Patients’ health-related quality of life measured using the EQ-5D-3L questionnaire at baseline, admission for surgery, and clinical discharge [43].
- Patients’ emotional status measured using the Positive and Negative affect Scale (PANAS) at baseline, admission for surgery, hospital discharge, and 2 weeks after surgery [44].
- Patients’ and caregivers’ mental well-being measured using the “Short Warwick Edinburgh Mental Well-Being Scale” (SWEMWBS) at baseline and 2 weeks after surgery [45].
- Patients’ and caregivers’ self-efficacy measured using the General Self-Efficacy in short form (GSE) questionnaire at baseline and 2 weeks after surgery [46].
- Patients’ activation status measured using the Patient Activation Measure short form (PAM-13) at baseline, admission for surgery, and 2 weeks after surgery [47].

All these questionnaires have proven to be reliable and effective in measuring the respective variables.

## Results

### Overview

**Figure 1:**
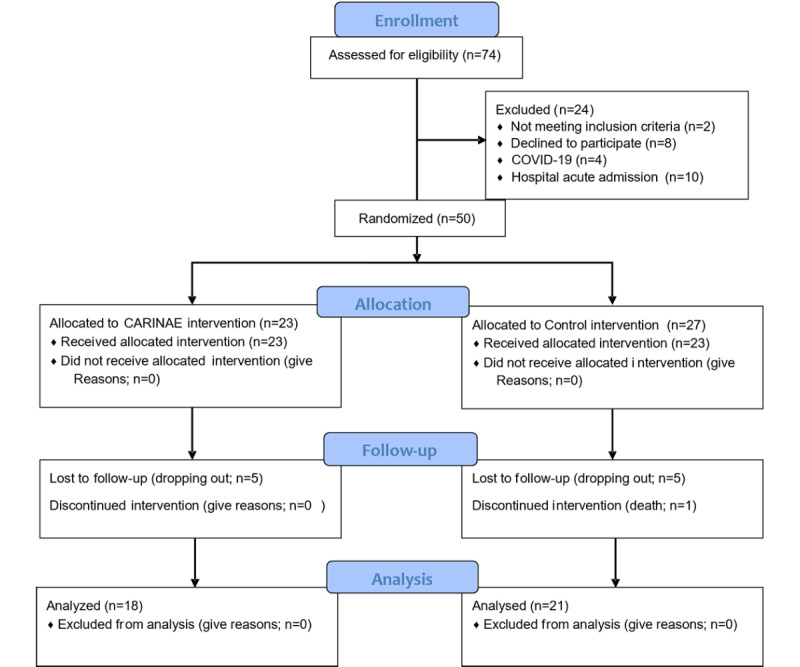
CONSORT (Consolidated Standards of Reporting Trials) diagram of the trial.

The diagram of the study is shown in Figure 1. From September 2021 to March 2022, a total of 74 patients were assessed for eligibility and 24 patients were excluded according to the following: (1) not meeting inclusion criteria (n=2); (2) declined to participate (n=8); (3) COVID-19 urgency (n=4); and (4) hospital acute admission (n=10). A total of 50 patients participated in the study, 23 patients were allocated to the CARINAE group (intervention group) and 27 participants were allocated to the control group. In total, 10 patients dropped out of the study (5 patients in the intervention group and 5 patients in the control group). In addition, 1 control group patient died during the surgery. A total of 39 patients finalized the trial and their data have been analyzed (21 patients in the control group and 18 patients in the intervention group; see the CONSORT [Consolidated Standards of Reporting Trials] flowchart in Figure 1 and CONSORT checklist in Multimedia Appendix 1).

The intervention group consisted of 18 patients (10 male and 8 female patients; mean age 45.38, SD 26.2, range 12-91 years) and the control group consisted of 21 patients (11 male and 10 female patients; mean age 56.66, SD 27, range 12-91 years). Furthermore, intervention group surgeries included 1 patient undergone coronary bypass, 1 cardiac valve replacement, 4 scoliosis, 2 hip and 2 knee replacements, 3 prostate cancer, 2 bladder cancer surgeries, and 3 maxillofacial surgery types (see Multimedia Appendix 2 for sociodemographic characteristics).

The control group consisted of 3 patients undergone cardiac valve replacement, 2 scoliosis, 2 hip replacements, 4 knee replacements, 2 prostate cancer, 4 bladder cancer, and 4 maxillofacial surgery types (see Multimedia Appendix 2 for sociodemographic characteristics). The baseline table highlights a potential bias with regard to the age of the participants in both intervention and control groups (ca. 10 years), see the impact of that in the Discussion/Limitations section.

In addition, 22 caregivers participated in the study (11 in the control group and 11 in the intervention group). A total of 5 caregivers dropped out of the study (4 in the control group and 1 in the intervention group). A total of 17 caregivers finalized the trial and their data have been analyzed. This difference in drop out might be resulting from the lack of incentive to stay in the control group.

Finally, 12 health care professionals answered the three questionnaires (VAS, SWEMWBS, and Caregiver GSE), and their data were analyzed; 5 health care professionals belonged to INRCA—Italy, 4 to the SAS—Cordoba, Spain, 2 to the HSJD—Barcelona, Spain and 1 to the Parc Taulí, Spain.

### Descriptive Statistics

Multimedia Appendix 2 presents descriptive statistics for the most important sociodemographic characteristics such as age, sex, digital expertise, educational level, type of medication, type of surgery, and previous comorbidities. Most of the participants had basic (n=13) or advanced digital skills (n=17) and 8 of them considered themselves experts. Regarding previous comorbidities, 11 of them had cancer in the past, 7 of them had cardiovascular disease, 1 had diabetes mellitus, 1 had renal disease, and 4 of them had mental diseases.

### Clinical Outcome Parameters

Regarding the clinical variables, descriptive statistical metrics along with results of univariate analysis between intervention and control groups by *t* test method are presented in Multimedia Appendix 3.

**Figure 2:**
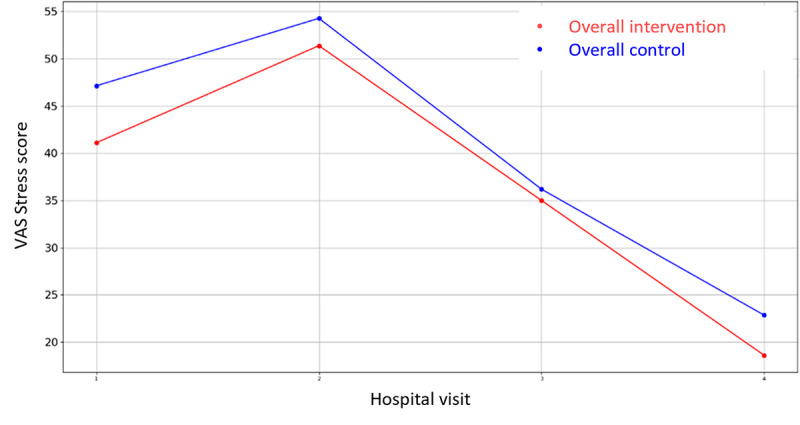
Evolution of mean VAS Stress score during the 4 hospital visits (initial visit, hospital, hospital discharge, and 14 days postsurgery). VAS: Visual Analogue Scale.

**Figure 3:**
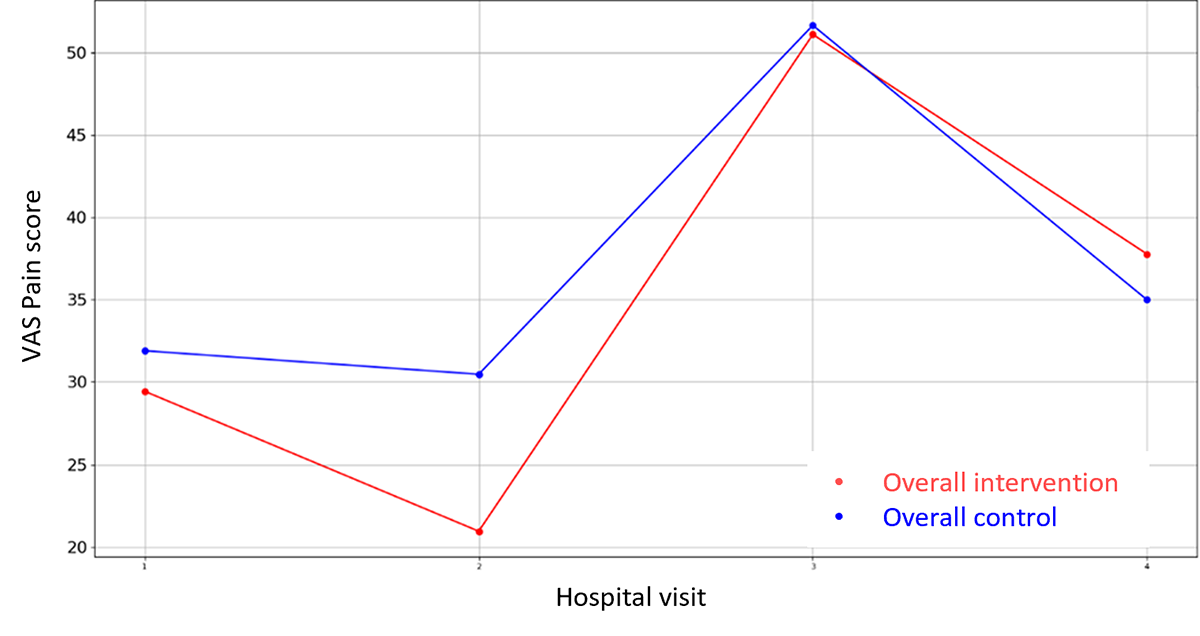
Evolution of mean VAS Pain score during the 4 hospital visits (initial visit, hospital, hospital discharge, and 14 days postsurgery). VAS: Visual Analogue Scale.

**Figure 4:**
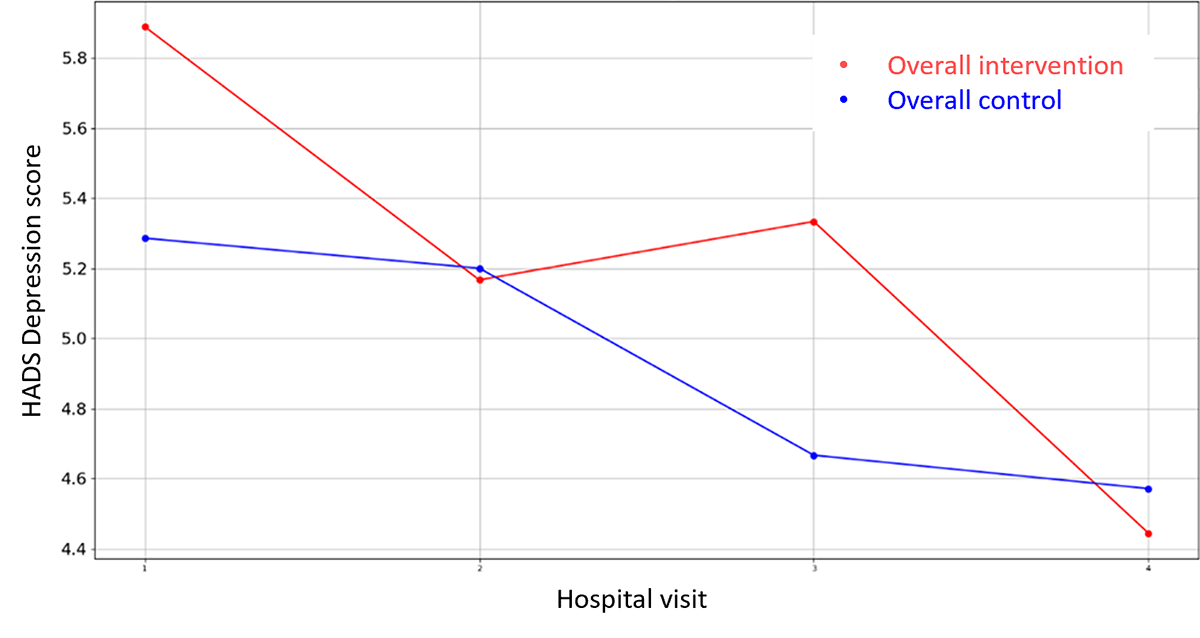
Evolution of mean HADS Depression score during the 4 hospital visits (initial visit, hospital, hospital discharge, and 14 days postsurgery). HADS: Hospital Anxiety and Depression Scale.

**Figure 5:**
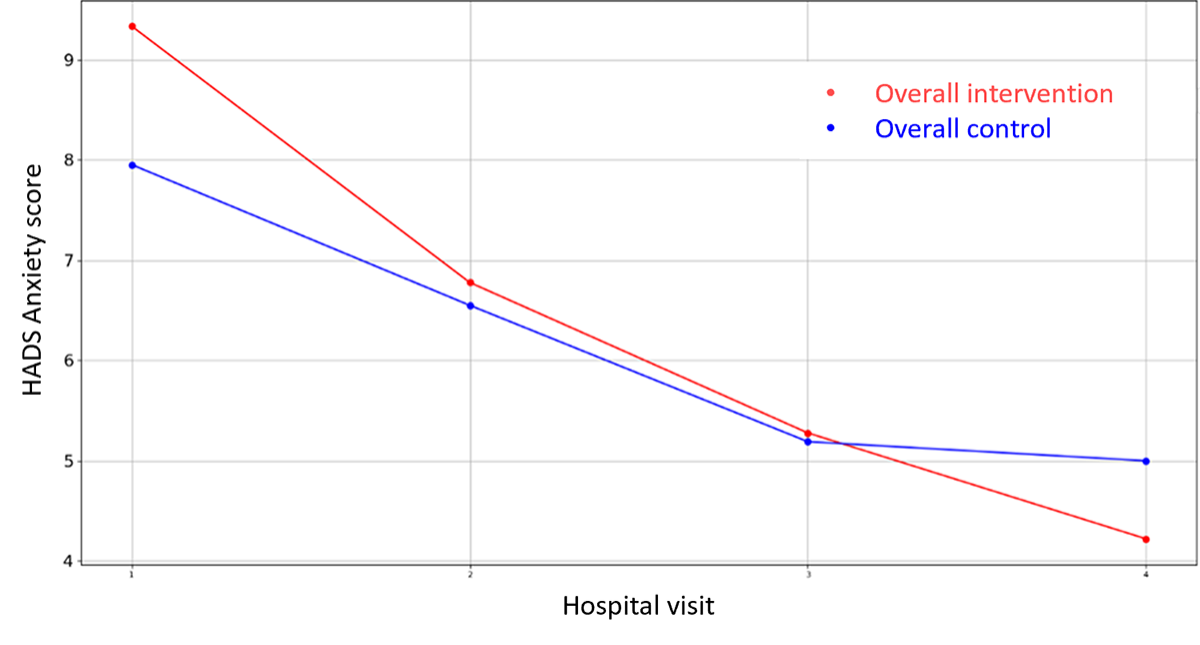
Evolution of mean HADS Anxiety score during the 4 hospital visits (initial visit, hospital, hospital discharge, and 14 days postsurgery). HADS: Hospital Anxiety and Depression Scale.

**Figure 6:**
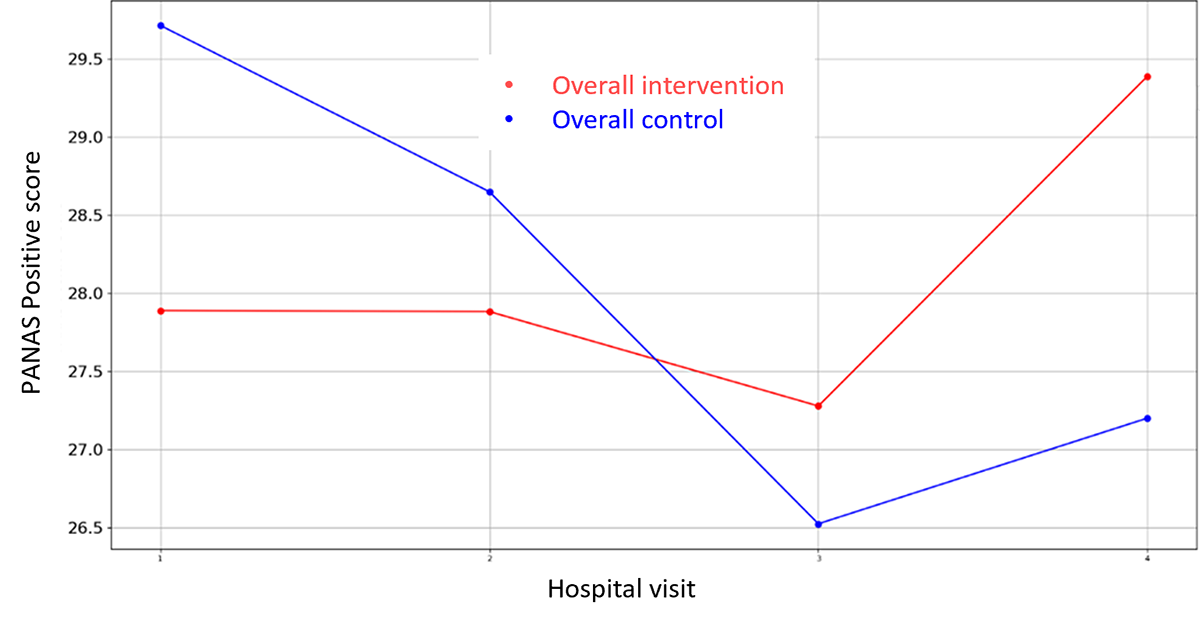
Evolution of mean PANAS Positive score during the 4 hospital visits (initial visit, hospital, hospital discharge, and 14 days postsurgery). PANAS: Positive and Negative Affect Scale.

**Figure 7:**
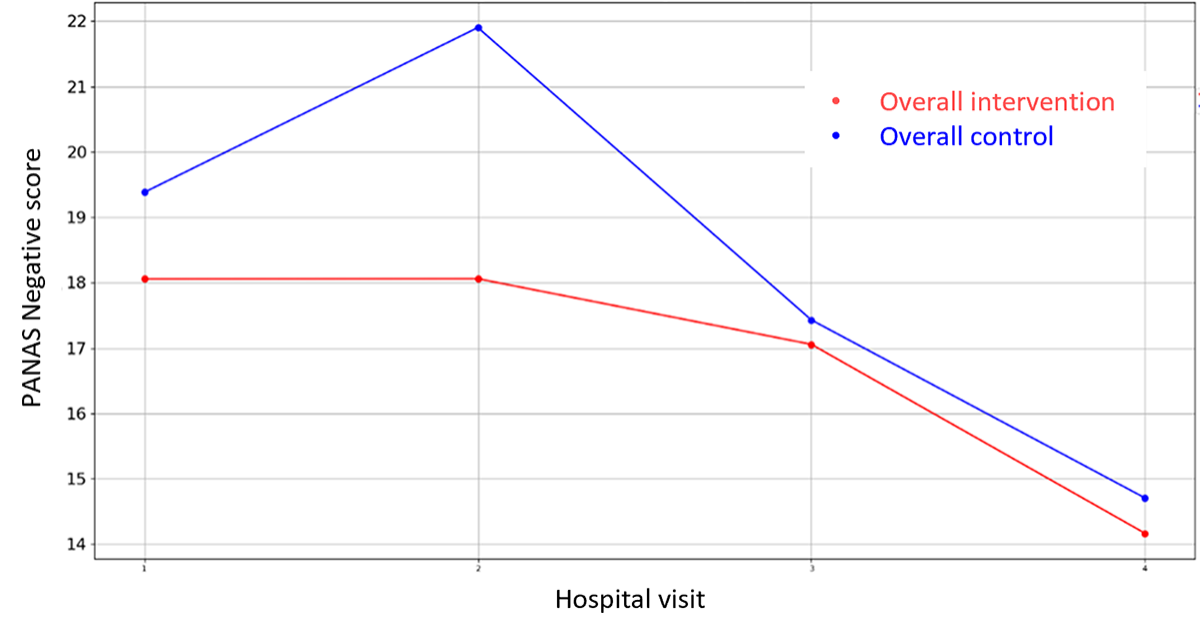
Evolution of mean PANAS (Positive and Negative Affect Scale) Negative score during the 4 hospital visits (initial visit, hospital, hospital discharge, and 14 days postsurgery).

Among all the variables in Multimedia Appendix 3, the VAS values for stress were lower for the intervention groups in all the overall cases (prehospitalization, hospitalization, and discharge) but they did not reach significance. There is only one that demonstrates statistical differences between control and intervention groups: HADS Depression Score during hospital admission in Parc Taulí. For the rest variables, the hypothesis of equal mean between intervention and control groups cannot be rejected due to the high *P* value outcome of the statistical tests. In addition, the mean VAS Stress, VAS Pain, HADS Depression, HADS Anxiety, PANAS Positive, and PANAS Negative score evolution throughout the 4 hospital visits follow-up period are illustrated in Figures 2-7.

**Figure 8:**
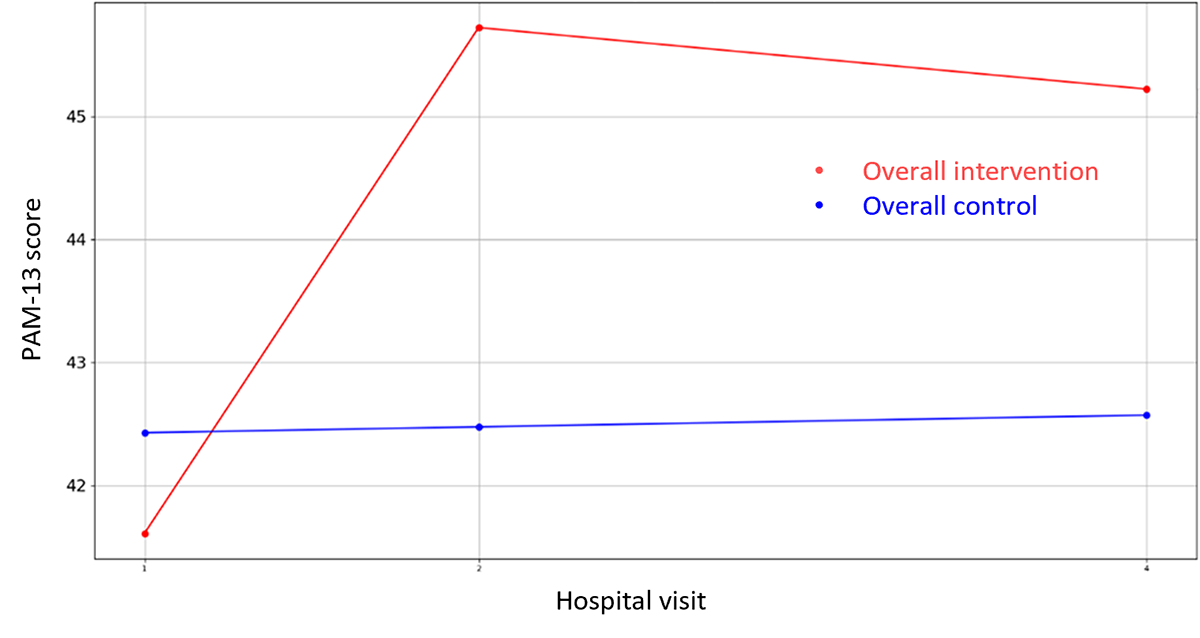
Evolution of mean PAM-13 score during the 4 hospital visits (initial visit, hospital, hospital discharge, and 14 days postsurgery). PAM-13: Patient Activation Measure 13.

**Figure 9:**
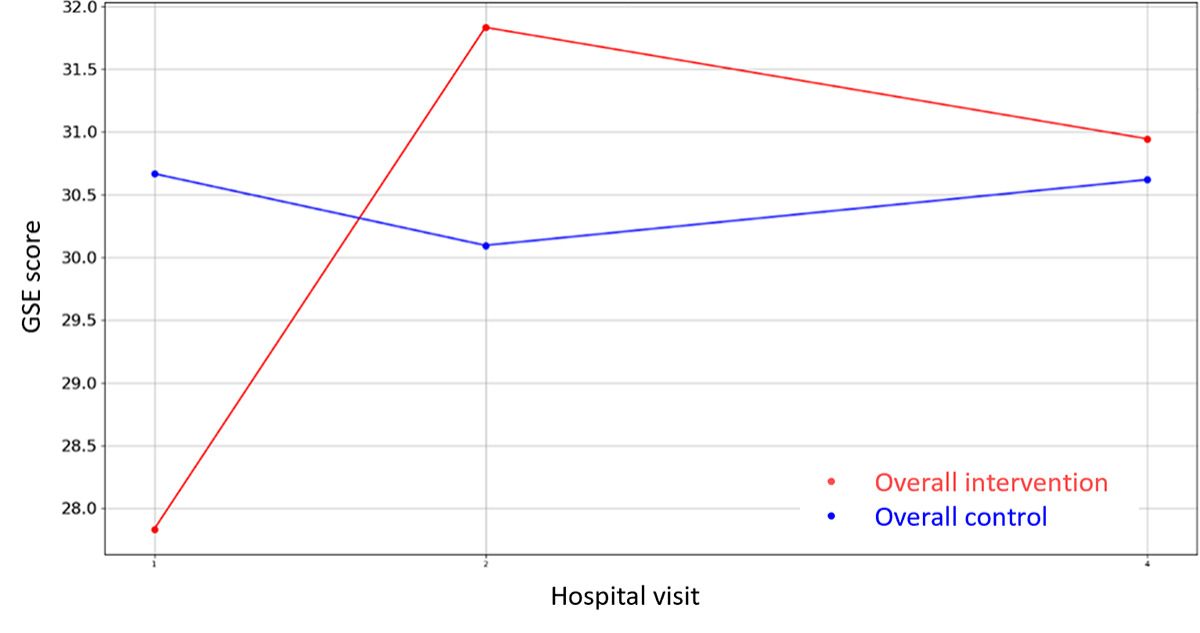
Evolution of mean GSE score during the 4 hospital visits (initial visit, hospital, hospital discharge, and 14 days postsurgery). GSE: General Self-Efficacy.

**Figure 10:**
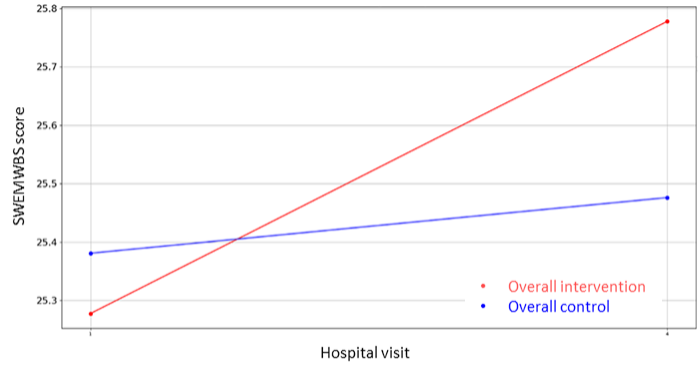
Evolution of mean SWEMWBS score during the 4 hospital visits (initial visit, hospital, hospital discharge, and 14 days postsurgery). SWEMWBS: Short Warwick Edinburgh Mental Well-Being Scale.

**Figure 11:**
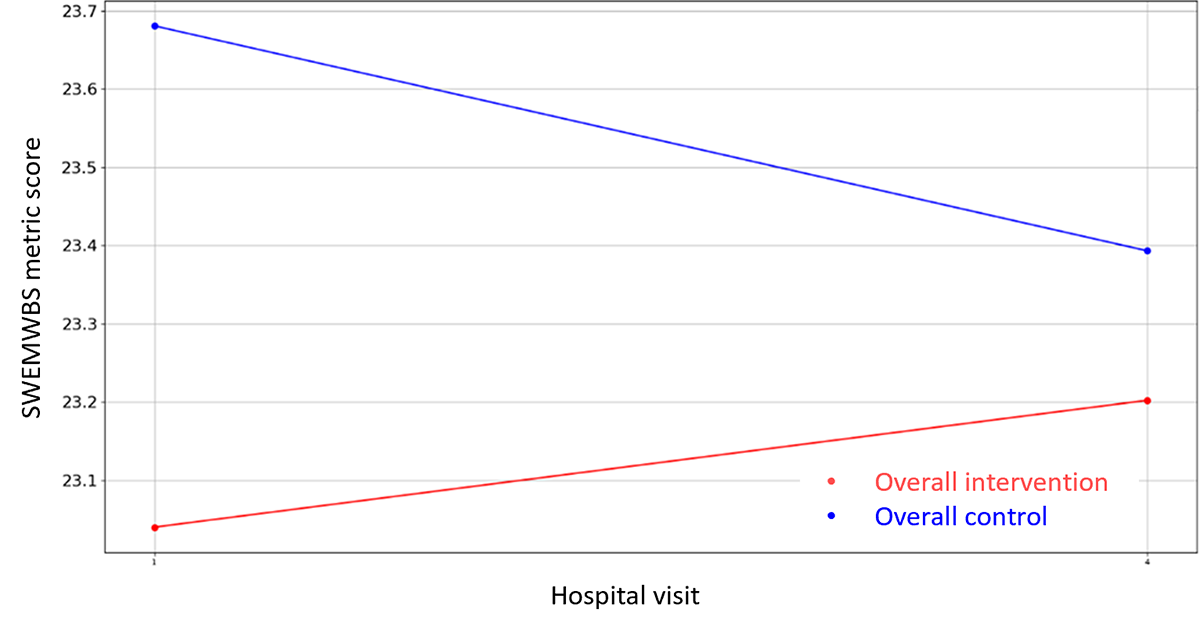
Evolution of mean SWEMWBS Metric score during the 4 hospital visits (initial visit, hospital, hospital discharge, and 14 days postsurgery). SWEMWBS: Short Warwick Edinburgh Mental Well-Being Scale.

**Figure 12:**
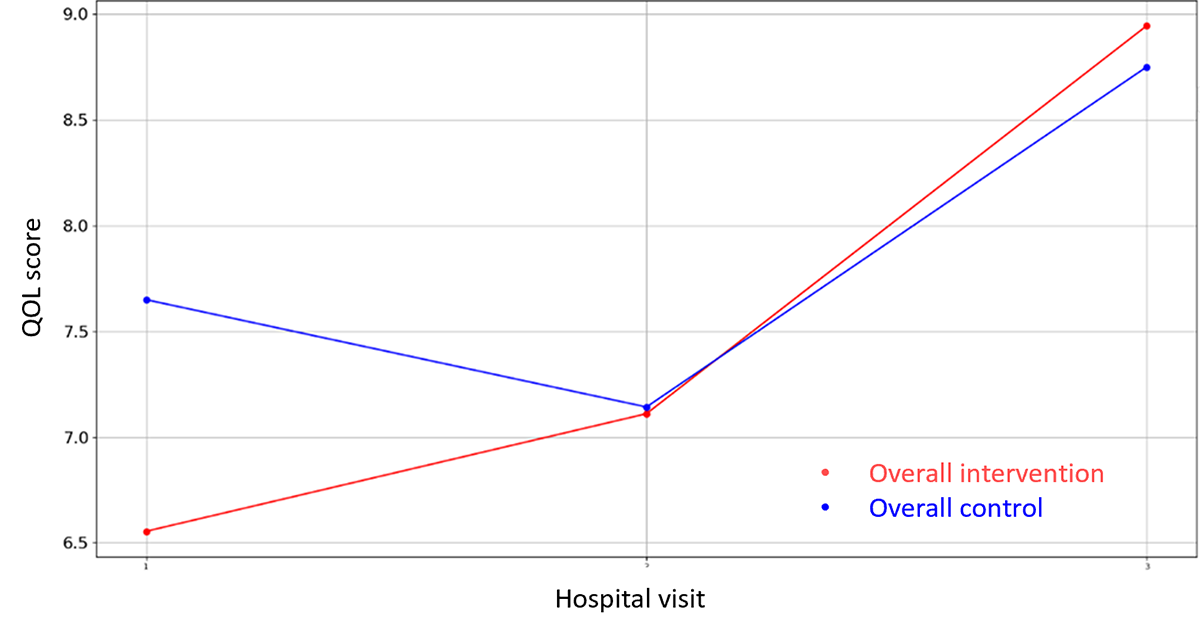
Evolution of mean QOL score during the 4 hospital visits (initial visit, hospital, hospital discharge, and 14 days postsurgery). QOL: Quality of Life.

**Figure 13:**
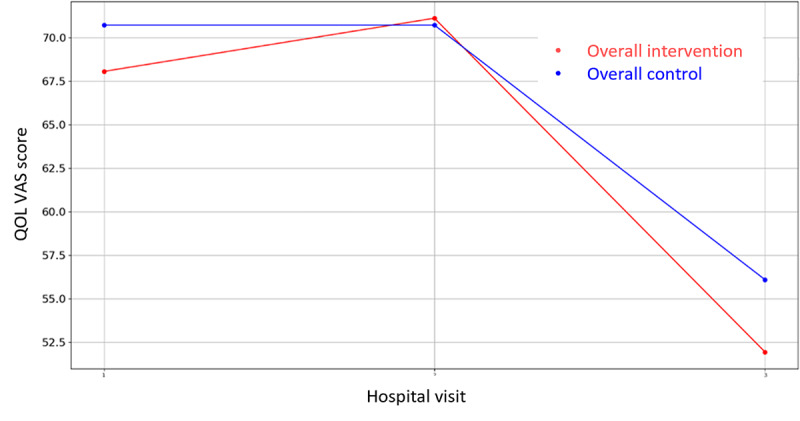
Evolution of mean QOL VAS score during the 4 hospital visits (initial visit, hospital, hospital discharge, and 14 days postsurgery). QOL: quality of life; VAS: Visual Analogue Scale.

**Figure 14:**
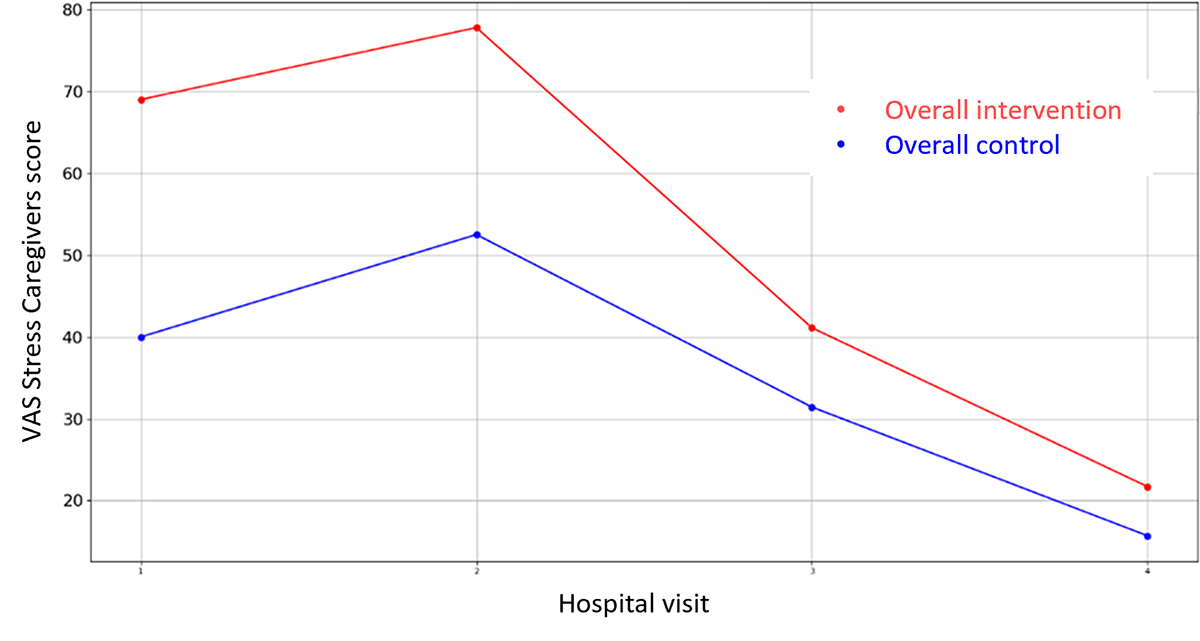
Evolution of Caregiver’s VAS Stress score during the 4 hospital visits (initial visit, hospital, hospital discharge, and 14 days postsurgery). VAS: Visual Analogue Scale.

**Figure 15:**
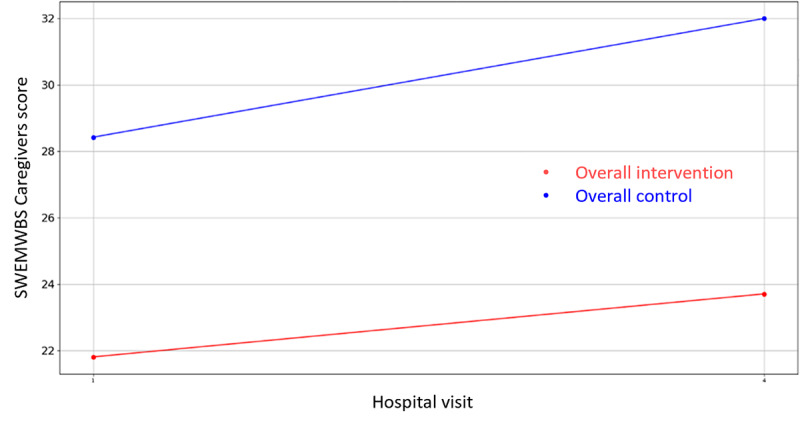
Evolution of Caregiver’s SWEMWBS score during the 4 hospital visits (initial visit, hospital, hospital discharge, and 14 days postsurgery). SWEMWBS: Short Warwick Edinburgh Mental Well-Being Scale.

**Figure 16:**
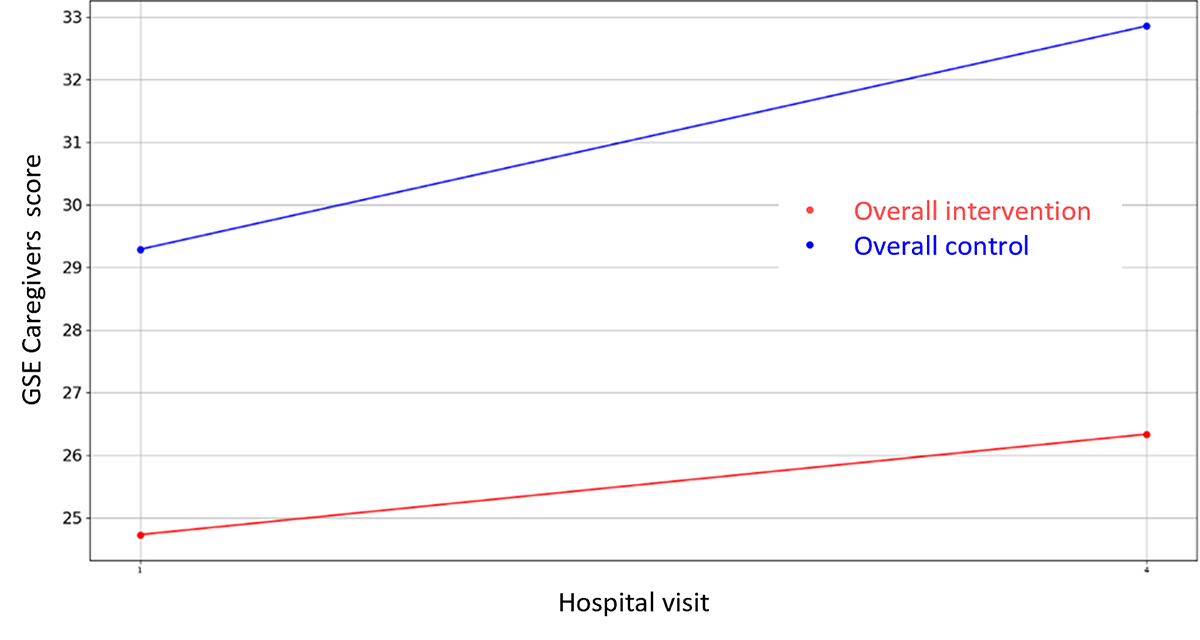
Evolution of Caregiver’s GSE score during the 4 hospital visits (initial visit, hospital, hospital discharge, and 14 days postsurgery). GSE: General Self-Efficacy.

Likewise, regarding the nonclinical outcome parameters of Multimedia Appendix 4, the same statistical methodology has been designed and executed to identify the most statistically significant variables. In this case, there are three variables where the hypothesis of equal means can be rejected (*P*<.05), that is, PAM-13 Hospital Admission item 13, VAS Stress—hospital discharge, GSE, and the corresponding questionnaires’ (PAM-13, GSE, SWEMWBS, QOLmean, VAS Stress Caregiver, SWEMWBS Caregiver, and GSE Caregiver) score evolution graphics are presented in Figures 8-16.

### Surgery’s Exploratory Analysis

#### ANOVA Analysis

This section aims to investigate any possible statistical effect of the type of surgery on the patients between the intervention and control groups. It uses the mixed-ANOVA statistical tests applied to each unique surgery type group. Thus, depending on the surgery, ANOVA results are as follows:

- Hip replacement surgery: *F*_29,29_=1.38; *P*=.19; η*_p_*^2^=0.58
- Knee replacement surgery:*F*_29,116_=259; *P*=.06; η*_p_*^2^=0.27
- Maxilofacial surgery: *F*_29,145_=0.67; *P*=.89; η*_p_*^2^=0.11
- Cardiac valve replacement surgery: *F*_29,58_=0.8, *P*=.73; η*_p_*^2^=0.28
- Bladder surgery: *F*_29,116_=0.38; *P*=.99; η*_p_*^2^=0.08
- Prostate surgery: *F*_29,87_=0.73; *P*=.82; η*_p_*^2^=0.19
- Scoliosis surgery: *F*_29,116_=0.13; *P*=.007; η*_p_*^2^=0.32

The *P* value obtained from ANOVA analysis in scoliosis is significant (*P*<.05), and therefore, we conclude that there are significant differences among intervention and control patients undergone that type of surgery.

#### Tukey Approach

For further investigation of the scoliosis data, an essential pair-wise correlational analysis has been implemented to specify the exact pair of variables whose statistics are significantly different between the intervention and control groups. One of the most commonly used post hoc tests is Tukey’s test, which allows us to make pairwise comparisons between the means of each group while controlling for the family wise error rate.

Thus, we would conclude that there is a statistically significant difference (*P*<.05) between the means of groups: VAS Stress (hospital discharge) – VAS Pain (postoperation day) VAS Stress (hospital discharge) – HADS Depression (enrollment) VAS Stress (hospital discharge) – HADS Depression (hospital admission) VAS Stress (hospital discharge) – HADS Depression (hospital discharge) VAS Stress (hospital discharge) – HADS Depression (postoperation day) VAS Stress (hospital discharge) – HADS Anxiety (enrollment) VAS Stress (hospital discharge) – HADS Anxiety (hospital admission) VAS Stress (hospital discharge) – HADS Anxiety (hospital discharge) VAS Pain (enrollment) – VAS Pain (postoperation day) VAS Pain (hospital discharge) – VAS Pain (postoperation day) VAS Pain (hospital discharge) – HADS Depression (enrollment) VAS Pain (hospital discharge) – HADS Depression (hospital admission) VAS Pain (hospital discharge) – HADS Depression (hospital discharge) VAS Pain (hospital discharge) – HADS Depression (postoperation day) VAS Pain (hospital discharge) – HADS Anxiety (enrollment) VAS Pain (hospital discharge) – HADS Anxiety (hospital admission) VAS Pain (hospital discharge) – HADS Anxiety (hospital discharge) HADS Depression (enrollment) – PAM (enrollment).

#### Linear Mixed Models

Apart from ANOVA tests, to identify any connections between the input data clustered by type of surgery, another statistical approach has been tested, the linear mixed modeling technique.

In this exploratory analysis VAS Stress, VAS Pain, HADS Depression, HADS Anxiety and PANAS questionnaires’ scores have been submitted to a linear mixed models (LMM) analysis wherein “time,” “group (intervention vs control),” their interaction (time × group), age, and sex have been included as fixed factors. On the other hand, “subjects” and “surgery” have been submitted as random factors. The most significant LMM model’s results appear to be the ones with dependent variables the questionnaire VAS Stress and VAS Pain. The corresponding results are presented in Tables 1 and 2.

**Table 1:** Results of the linear mixed models for the VAS^a^ Stress.

	Coefficient	SE	*P* value
Hospital admission score	8.59	5.7	.13
Hospital discharge score	–8.72	5.7	.13
Postoperation visit score	–23.46	5.7	<.001
Sex (female)	11.92	5.06	.02
Groups (intervention/control)	–8.99	4.616	.05
Age	–1.58	0.448	.001

^a^VAS: Visual Analogue Scale.

**Table 2:** Results of the linear mixed models for the VAS^a^ Pain.

	Coefficient	SE	*P* value
Hospital admission score	–4.69	5.195	.36
Hospital discharge score	20.64	5.195	<.001
Postoperation visit score	5.513	5.195	.29
Sex (female)	0.85	4.6	.85
Groups (intervention/control)	–2.22	4.2	.59
Age	–0.28	0.349	.42

^a^VAS: Visual Analogue Scale.

#### Feature Importance Analysis

Feature importance refers to techniques that calculate a score for all the input features for a given model—the scores simply represent the “importance” of each feature. A higher score means that the specific feature will have a larger effect on the model that is being used to predict a certain variable. The following figures correspond to the computation of the most important factors linked to the principal questionnaires of this study (VAS Stress, VAS Pain, HADS Depression, HADS Anxiety, PANAS) using the XGBoost Regressor. The results are shown in the Multimedia Appendices 5-10.

### Adherence and Engagement Analysis to CARINAE App

Adherence to VAS Pain and Stress, as well as to Wound Healing questionnaires into the CARINAE app have been analyzed to show the actions performed by the patients and the results show an overall adherence on VAS Pain of 47.23%, on VAS Stress of 68.41%, and Wound Healing of 2.5%. In the following table (Table 3), the adherence rates are also presented per hospital.

Further, the engagement table to the CARINAE app is presented in Table 4 for the various hospitals showing the total amount of interactions and patient sessions.

**Table 3:** Mean adherence to VAS^a^ Pain, VAS Stress, and Wound Healing questionnaires into the CARINAE app.

Hospital	Days in hospital	Days in study	Completed VAS Pain	VAS Pain rate	Completed VAS Stress	VAS Stress rate	Wound Healing rate
INRCA^b^	3	33	23.33	73%	23	71.13%	3%
Parc Taulí	4	37	12.33	24.15%	15.6	30.50%	0%
Hospital Sant Joan de Déu	2.2	23.4	9.2	40.40%	20.2	79%	4.1%

^a^VAS: Visual Analogue Scale.

^b^INRCA: Instituto di Ricovero e Cura per Anziani.

**Table 4:** Engagement to CARINAE app in terms of patient and caregiver interactions, number of sessions, and session duration.

Hospital	Patients total interactions	Caregivers total interactions	Patients session duration (in seconds), mean (SD)	Caregivers session duration (in seconds), mean (SD)	Patients total number of sessions	Caregivers total number of sessions
INRCA^a^	526	N/A^b^	4.402 (132)	N/A	375	N/A
Parc Taulí	46	14	1.789 (54)	76 (22)	136	10
Hospital Sant Joan de Déu	264	43	3.456 (104)	2.143 (64)	190	167
SAS	34	N/A	87.683 (2630)	N/A	8	N/A

^a^INRCA: Instituto di Ricovero e Cura per Anziani.

^b^N/A: not applicable.

## Discussion

### Principal Findings

The main aim of this study was to test the feasibility and efficacy of a personalized stress and anxiety patient empowerment DH solution (CARINAE) on patients undergoing surgery compared to a control group. Furthermore, this study aimed to show the impact of the CARINAE solution on quality of life, emotional status, mental well-being, and self-efficacy, activation status on patients’ knowledge, skills, and confidence for self-management. Besides, the CARINAE solution has been assessed by health care professionals and patients and caregivers allocated to the intervention group, in terms of engagement.

According to our results, at baseline, patients of both groups reported generally normal levels of pain and stress, with higher levels of stress. Patients undergoing coronary bypass and cardiac valve replacement surgeries indicated higher levels of stress before the surgery (VAS>80), followed by hip and knee replacement surgeries in both pain and stress (VAS>60) compared to the other patients’ surgeries. Similar stress values can be observed by caregivers. Regarding depression, patients of both groups reported general normal levels but they indicated to experience anxiety (HADS score between 7 and 10). In detail, depression is primarily observed by older patients undergoing prostate and bladder cancer removal surgery, with values between the abnormal range (HADS depression abnormal range 11-21) and secondarily, with borderline values (HADS depression borderline range 8-10), by patients undergoing coronary bypass and cardiac valve replacement surgeries. In contrast, anxiety is more generalized among various patients, and the results showed that older patients undergoing an operation to remove prostate or bladder cancer report very high values, in the abnormal range (HADS anxiety >11), followed by the youngest undergoing scoliosis and maxillofacial surgeries (HADS anxiety=10), and hip and knee surgeries, as well as coronary bypass and cardiac valve replacement surgeries with borderline levels of anxiety (HADS anxiety borderline range 8-10). Regarding positive and negative affect scores, patients of both groups showed normal levels of positive affect (mean scores normal levels 33.3, SD 7.2) and higher values regarding negative affect (mean scores normal levels 17.4, SD 6.2). Regarding patients’ knowledge, skills, and confidence for self-management, no patient of either group found it important to take action with respect to the situation and the self-efficacy perception resulted quite high in most patients and caregivers (GSE>25). Finally, with respect to mental well-being and quality of life, at baseline patients and caregivers of both groups present quite high rates of mental well-being and quality of life. With respect to the latter two measures, it can be emphasized that patients who are older and have undergone more delicate surgeries, such as coronary bypass and cardiac valve replacement surgeries showed lower levels than other surgeries and age. A potential bias is the difference in age between the control and intervention groups, the control group has on average 10 years more. In a small sample, this bias might have occurred inevitably, but it might be indicative of a selection bias. In future studies, we should consider the possibility of including a “control intervention” that also requires the use of some simple mobile-based technology (eg, watching educational videos on YouTube).

Looking at the relationship between the variables, overall patients show most strong associations between negative emotions, anxiety, and stress, especially in the preoperative phase (recruitment and hospital admission), which in some cases are also maintained in the postoperative phase (hospital discharge and postoperative follow-up visit), but nevertheless, the presence of a strong inverse relationship between mental well-being and anxiety at the postoperative follow-up visit suggests that as anxiety decreases, patients’ mental well-being increases again.

Examining the 2 groups separately, the control group shows greater relationships between the more negative psychological dimensions, such as anxiety, stress, negative emotions, and depression, whereas the intervention group shows more positive relationships between the psychological dimensions of self-efficacy, self-management, and mental well-being, suggesting that CARINAE solution could have a positive effect and impact on the reduction of stress and negative emotions. In detail, the control group showed levels of stress, depression, anxiety, and negative emotions that remained constant throughout the entire perioperative process, and only at the end of the process could changes in the perception of greater mental and physical well-being be observed. There were positive trends in the intervention group, although not significant from a statistical point of view. The intervention group shows a greater perception of mental well-being, self-efficacy, and self-management during the entire perioperative process and not only at the end of the process. Considering the clinical outcome parameters on pain, stress, anxiety, and depression, a significant difference between the two groups has been found in the depression subscale regarding the hip and knee surgeries, still showing values within the normal range. Although no other statistically significant differences were found, it can be seen how the values with respect to pain remain low at the time of hospitalization and how they increase after the operation and then decrease again at the follow-up visit. Pain is known to be related to stress levels. The highest postoperative pain values are perceived more in those patients who underwent a more invasive operation (eg, coronary bypass and cardiac valve replacement surgeries) than in those whose operation was less invasive (prostate and bladder cancer). As expected, as far as stress was concerned, no significant differences were found, but the values increased at hospital admission, compared to baseline, and these values decreased at discharge and more so at the follow-up visit. Looking at the individual operations, the cardiac valve replacement surgeries and hip and knee replacement surgeries on admission to the hospital are the ones that cause the most stress for patients. Finally, the values of anxiety and depression remained within the normal ranges, and with respect to the baseline, it can be observed that in patients undergoing prostate or bladder cancer removal surgery who had high levels, the levels of both parameters steadily decreased at various stages of the perioperative process. We should notice that the project and the intervention aim at reducing stress levels, and not clinical anxiety disorder or major depression. This is quite important since a narrower focus on major mental health disorders might reduce the applicability of the intervention to most of the population who undergoes surgery. We should be aware that the exploratory analyses might, due to multiple testing, be subject to type 1 error, and cautious interpretation of the analysis is warranted.

Regarding the nonclinical outcome parameters, no significant differences have been found. However, the positive effect values remain stable and in the normal range during the perioperative process, increasing more after the hospital discharge, while negative values decrease after the operation and at the follow-up visit. Regarding patients’ knowledge, skills, and confidence for self-management, no patient of either group found it important to take action with respect to the situation, and the self-efficacy perception resulted higher in patients and caregivers after the surgeries than the baseline (GSE>30). Finally, with respect to mental well-being and quality of life, in the follow-up visit, patients and caregivers of both groups presented higher rates of mental well-being and lower rates of quality of life than baseline, suggesting a faster recovery process of mental well-being in terms of recovery of feeling relaxed, optimistic, thinking clearly, and dealing with problems well and a slower recovery of quality of life in terms of recovery of mobility, autonomy, and usual activities. These results are not conclusive, and a bigger study might be needed to identify the level of effectiveness of the intervention. However, major aspects deserve further attention prior to a larger study. The low awareness about the importance of self-management might be an indicator of low health literacy levels. It might be wise to consider strategies that as part of the intervention address awareness of lifestyle factors for recovery even before the intervention itself.

The founded differences between the two groups may be linked to the young age of the participants and consequently to the related hormonal and neurodevelopment changes. Indeed, these changes are currently conceptualized in terms of imbalance between systems supporting reactivity and regulation, specifically nonlinear changes in reactivity networks and linear changes in regulatory networks [48].

In addition, as identified by LMMs, stress and pain showed other statistical differences in accordance with other parameters, showing that experiences of stress seem to differ according to age and sex. The level of stress rises with aging and is higher in female patients than in male patients. In addition, both stress and pain also differed depending on the phase of the perioperative process in which patients find themselves. In detail, pain presented more effect and impact at hospital discharge, showing the highest levels of the perioperative process, and stress at the end of the entire process (at follow-up visit), showing the lowest levels of the perioperative process. Further analyzing the importance of the various features using XGBoost revealed that stress, anxiety, pain, and negative affect measures are interrelated as expected and that physical activity is also important.

Finally, according to the adherence to the psychometric questionnaires in the CARINAE app, we observed that younger and older participants were more involved in the self-evaluation regarding stress with 79% and 71%, respectively. On pain, older participants were involved more than younger with 73% of responses by the older and 40% by the younger. These data are also reflected in the use of the CARINAE app, where the greatest interactions were found among older and younger patients. These data suggest, on the one hand, a greater provision of time by older people, and on the other hand, extensive use of technology by younger people and overall high involvement in the use of CARINAE for both ages.

### Comparison to Literature

Although no significant differences have been found between groups, advances in DH interventions are playing great support in enhancing awareness about health conditions and for the management of mental health by relying on both nonimmersive (eg, such as mobile apps) and immersive systems (eg, VR) [16]. These include support for patients in the management of anxiety, stress [37], and pain [18]. On one hand, mobile apps for perioperative processes are becoming a hot topic, allowing to provide psychoeducational contents, mental well-being activities to reduce pain and stress, up-to-date information, tracking personal health data, reminding and engaging patients, and communicating in a cost-effective way. On the other, VR has been used in multiple health care applications including reducing stress and pain, training medical practitioners, patient counseling, cognitive rehabilitation, physical therapy in medicine, and for diagnostic and treatment needs in dentistry, mental health management, and surgery [19]. A multiuser immersive VR system was developed and used during presurgical discussions in a prospective patient cohort undergoing cerebrovascular surgery [20]. An immersive VR intervention adopted in pediatric patients to manage pain and anxiety provided a new, easy, and cost-effective intervention that can be applied to other painful and stressful medical procedures [49]. Pain is a highly distressing symptom for patients in all clinical settings and stress and anxiety levels influence it. VR applications have proven to be efficient in stress relief and pain management, mainly due to their distractive properties [22].

In the literature, there is a lack of understanding of the feasibility of combining VR with mobile-based technologies as the main channel of the provision of a DH intervention [36]. In this study, what is explored is the feasibility of a DH intervention that leverages the latest mobile and VR technologies within the use case of helping patients manage stress and anxiety during surgery while promoting healthy recovery.

The findings of this project, reveal the importance of addressing this type of intervention as a service design approach with a strong focus on implementation aspects. A recent guideline by the World Health Organization in DH reinforces the importance of addressing training and supporting the environment as key success aspects in DH implementations [50]. In addition, infrastructure was a key element (eg, connectivity issues). The workforce is not only crucial from a service delivery point of view, but also a key channel to facilitate patient DH literacy which in this very complex setup with multiple devices and features is especially important.

### Strengths and Limitations

Several strategies and techniques have been proposed to manage preoperative stress and anxiety that can be effective in supporting patients to cope with a wide range of stressful health situations. In the current health care settings, however, it is not very common to provide patients with stress and anxiety relief support prior to a surgical procedure. Usually, VR-enhanced solutions focus only on providing informative content, neglecting the importance of patient empowerment with a more robust educational curriculum. This study has been among the first to evaluate the potential effectiveness of a comprehensive technology in reducing perioperative stress and anxiety. CARINAE provided a unique combination of endpoints and the integration of knowledge deriving from several domains, including stress or anxiety management, patient empowerment, communication with medical professionals, adaptation to illness, self-regulation and self-management, and adaptation to medical procedures. The study has been successful in terms of identifying the key impacts of such type of intervention and provided enough exploratory insights to redesign the intervention and establish a new randomized controlled trial design to provide more conclusive data on efficacy. At the same time, this study allowed us to identify implementation issues that need to be addressed prior to larger studies and implementations. For example, larger trials with more participants are advisable for future work, which will facilitate the application of ANCOVA analysis.

This solution allowed participants to receive constant feedback to improve their appraisal and coping skills in an entertaining and motivating manner. It focuses on patient empowerment through active participation in the process and is dynamically adapted according to operation type, patient preferences, needs, and medical history all the way through the preclinical phase, admission, and discharge, in a continuous and personalized way. At the same time, it facilitated effective interactions between patients and health care professionals, through user-friendly and intelligent communication. It used the spaced learning methodology to help patients understand and learn the diverse aspects of their surgical process, from presurgery requirements to recovery steps with stress management all along the process, and provides multichannel anxiety and stress relief personalized content.

CARINAE solution, combining mobile health and VR technologies with a web app, provided positive preliminary results in reducing perioperative stress and creating effective collaborations between physicians or surgeons and their patients whilst supporting them in improving their knowledge in related domains. CARINAE has shown the potential to improve physical and emotional reactions to a stressor, such as surgical operations, to increase the levels of calmness to promote a sense of well-being and to empower patients in preoperative conditions. Information provided through the platform advances and enhances health literacy and digital competence and increases the participation of the patient in the decision-making process. Integration with third-party applications has facilitated the exchange of important information between patients and physicians, as well as between personal applications and clinical health systems.

While the findings of this study are interesting and valuable, it has some limitations. First, the small sample size, as well as the high variability of the ages and surgeries might limit the generalizability of the results. Given the pragmatic nature of this feasibility study, its small sample size was anticipated, further compounded by emergent challenges amid the COVID-19 crisis. The study did not aim to have a conclusive answer in terms of effectiveness, but to gather insight on whether it can be done as suggested in related literature [51]. As such, this study provides insights to support the design of a future randomized controlled trial. While a prospective observational design was considered, its susceptibility to confounding due to temporal changes prompted the inclusion of a control group, albeit with a heightened risk of limited significance. Moving forward, future research should prioritize more robust, adequately powered clinical studies to offer comprehensive insights.

Furthermore, pain and anxiety management guidelines might differ from one clinical setting to another, thus providing limited evidence based on raw cross-comparisons between different health care providers. Finally, the different durations of each surgery and each process limited the engagement data to the DH solution. Third, no data about use have been gathered from the VR app, limiting the quantitative performance rates and engagement to the VR component.

### Recommendations for Future Research, Development, and Clinical Practice

An area of future improvement that both this feasibility study and other DH solutions demonstrate the need for is long-term patient engagement. Indeed, according to the literature, as mobile health apps grow more prevalent, many have low attrition rates, reducing the relevance of data collection for the study [51]. Potential hurdles to long-term involvement include the patients’ functional condition and the workload associated with performing in-app tasks. Our findings are aligned with those studies. This is especially true for procedures such as cancer and cardiac surgeries, where the target population is older and potentially more functionally constrained than younger patient populations. To address the issue of long-term engagement, potential solutions include developing even more simplified versions of the solution that are easier to use for those unfamiliar with technology, using SMS text messaging–only modes of communication, and using shorter surveys that take less time to complete. For younger patients, on the contrary, more videos and images instead of text could engage more. Additionally, it may empower patients further if they are shown the outcomes of their data and are able to visualize and quantify in real-time, how their condition has improved. Previous studies have noted that patients provided positive feedback when presented with their study results and often noted reluctance to participate because they had not received personalized feedback from their care teams in previous app-based studies [51]. In this study, differences across sites might be due to differences in populations but also on how the solutions were promoted or introduced in each site. When integrating new technology into any health care setting, provider input may significantly streamline the integration of applications into already complex clinical workflows. Continually enhancing the experience and usability, from both the patient and provider perspectives, will allow the realization of the full potential of DH solutions for patients undergoing interventional procedures.

### Conclusions

We evaluated the feasibility of using CARINAE, a comprehensive DH solution, for patients undergoing surgery. This study examined the user experience and engagement with the solution, as well as the ability to collect patient-reported outcomes. Our results provide an important first step toward a deeper understanding of optimizing DH solutions to support patients undergoing surgery and for potential applications in remote patient monitoring and communication. This study highlighted how DH may be integrated into major procedures to improve patient education, emotional management, and engagement, and serve as a versatile clinical and research tool. Future studies will aim to test CARINAE in a larger cohort to establish its potential impact on health care resource utilization.

## Appendix

Multimedia Appendix 1 The completed CONSORT (Consolidated Standards of Reporting Trials) checklist.

Multimedia Appendix 2 Baseline characteristics of the control and intervention arms, both overall and per hospital.

Multimedia Appendix 3 Clinical outcome parameters of the control and intervention arms, both overall and per hospital.

Multimedia Appendix 4 Non-clinical outcome parameters of the control and intervention arms, both overall and per hospital.

Multimedia Appendix 5 HADS (Hospital Anxiety and Depression) Depression Feature Importance Analysis by XGBoost.

Multimedia Appendix 6 Panas Positive Feature Importance Analysis by XGBoost.

Multimedia Appendix 7 PANAS Negative Feature Importance Analysis by XGBoost.

Multimedia Appendix 8 VAS Pain Feature Importance Analysis by XGBoost.

Multimedia Appendix 9 HADS Anxiety Feature Importance Analysis by XGBoost.

Multimedia Appendix 10 VAS Stress Feature Importance Analysis by XGBoost.

## Abbreviations

CONSORT: Consolidated Standards of Reporting Trials
DH: digital health
GSE: General Self-Efficacy
HADS: Hospital Anxiety and Depression
INRCA: Instituto di Ricovero e Cura per Anziani
LMM: linear mixed model
PAM-13: Patient Activation Measure 13
PANAS: Positive and Negative Affect Scale
SAS: Hospital Universitario Reina Sofia
SJD: Sant Joan de Déu Hospital
SWEMWBS: Short Warwick Edinburgh Mental Well-Being Scale
VAS: Visual Analogue Scale
VR: virtual reality

## Data Availability

The datasets generated during or analyzed during this study are available from the corresponding author upon reasonable request.
