# Correction: Stress Reduction in Perioperative Care: Feasibility Randomized Controlled Trial

**DOI:** 10.2196/73608

**Published:** 2025-04-07

**Authors:** Haridimos Kondylakis, Irene Alice Chicchi Giglioli, Dimitrios Katehakis, Hatice Aldemir, Paul Zikas, George Papagiannakis, Santiago Hors-Fraile, Pedro L González-Sanz, Konstantinos Apostolakis, Constantine Stephanidis, Francisco J Núñez-Benjumea, Rosa M Baños-Rivera, Luis Fernandez-Luque, Angelina Kouroubali

**Affiliations:** 1 FORTH-ICS Heraklion Greece; 2 Adhera Health Inc Santa Cruz, CA United States; 3 ORamaVR SA Heraklion Greece; 4 Innovation & Data Analysis Unit Institute of Biomedicine of Seville IBiS/Virgen Macarena University Hospital/CSIC/University of Seville Seville Spain; 5 Polibienestar Institute University of Valencia Valencia Spain

In “A Stress Reduction in Perioperative Care: Feasibility Randomized Controlled Trial” (J Med Internet Res 2025;27:e54049) the authors noted one error.

Affiliation 2 has been revised from:

Adhera Therapeutics (United States), Wake Forest, CA, United States

to:

Adhera Health Inc, Santa Cruz, CA, United States

Additionally, the Conflicts of Interest section has been updated from:

IACG, HA, SH-F, and PLG-S are employees of Adhera Therapeutics, which owns the Adhera CARINAE DH Program. LF-L and SH-F were co-founders of Salumedia Labs, which was acquired by Adhera Therapeutics. All other authors declare no conflicts of interest.

To:

IACG, HA, SH-F, PLG-S, and LF-L are employees of Adhera Health Inc, which owns the intervention program described in this manuscript. All other authors declare no conflicts of interest.

The corrections will appear in the online version of the paper on the JMIR Publications website, together with the publication of this correction notice. Because this was made after submission to PubMed, PubMed Central, and other full-text repositories, the corrected article has also been resubmitted to those repositories.

