# Supplementary material for: Stress Reduction in Perioperative Care: Feasibility Randomized Controlled Trial
Source: J Med Internet Res. 2025 Jan 7;27:e54049. doi: 10.2196/54049 (PMC11751654; doi:10.2196/54049)
Supplement: Multimedia Appendix 2 [file jmir_v27i1e54049_app2.docx]

| Patients | Overall | | Hospital HSJD | | Hospital Parc Taulí | | Hospital SAS | | Hospital INRCA | |
| --- | --- | --- | --- | --- | --- | --- | --- | --- | --- | --- |
|  | Control | Intervention | Control | Intervention | Control | Intervention | Control | Intervention | Control | Intervention |
| N | 21 | 18 | 6 | 7 | 6 | 4 | 3 | 2 | 6 | 5 |
| **Age** | | | | | | | | | | |
| Mean±SD | 56.66±27 | 45.38±26.2 | 16.83±4.49 | 15.5 ±1.76 | 66.83±3.66 | 62±11.13 | 76.66±2.51 | 51.5 ±12.02 | 76.33±12.37 | 65.75±3.59 |
| Median (min-max) | 65 (12-91) | 56 (12-91) | 17.5 (12.0 - 24.0) | 16 (12-17) | 66.0 (62.0 - 72.0) | 60 (52-74) | 77 (74-79) | 51.5 (43-60) | 77.0 (61.0 - 91.0) | 66.5 (61 - 69) |
| **Sex** | | | | | | | | | | |
| Male N (%) | 11 (52.38) | 10 (55.55) | 4 (66.66) | 4 (57.14) | 3 (50) | 3 (75) | 2 (66.66) | 2 (100) | 4 (66.66) | 4 (80) |
| Female N (%) | 10 (47.62) | 8 (44.44) | 2 (33.33) | 3 (42.85) | 3 (50) | 1 (25) | 1 (33.33) |  | 2 (33.33) | 1 (20) |
| **BMI** | | | | | | | | | | |
| Mean±SD | 24.51±5.46 | 23.3 ±5.66 | 18.7 ±1.7 | 20.5 ±2.53 | 30.4 ±1.14 | 26.33±6.35 | 30.39±6.52 | 17.8 ±NaN | 22.71±2.86 | 25.05±5.41 |
| Median (min-max) | 24.51 (16.7-35) | 21 (17.8-35) | 19.9 (16.7-20) | 19.5 (18.3-25) | 30 (29-32) | 30 (19-30) | 30.39 (25.78 -35) | 17.8 (17.8-17.8) | 23.01 (19.0 - 26.23) | 24.88 (19 - 31.44) |
| **Educational level (ISCED)#** | | | | | | | | | | |
| N (%) |  |  |  |  |  |  |  |  |  |  |
| 0 early childhood | 2 (9.52) | 0 | 0 | 0 | 0 | 0 | 2 (66.66) | 0 | 0 | 0 |
| 1 primary | 5 (23.81) | 2 (11.11) | 1 (20) | 0 | 0 | 2 (66.66) | 1 (33.33) | 0 | 2 (40) | 0 |
| 2 lower secondary | 4 (19.04) | 2 (11.11) | 1 (20) | 1 (16.66) | 2 (40) | 0 | 0 | 0 | 1 (20) | 0 |
| 3 upper secondary | 3 (14.28) | 7 (38.88) | 0 | 2 (33.33) | 0 | 1 (33.33) | 0 | 0 | 2 (40) | 3 (75) |
| 4 post-secondary | 2 (9.52) | 2 (11.11) | 1 (20) | 1 (16.66) | 1 (20) | 0 | 0 | 0 | 0 | 0 |
| 5 short-cycle tertiary | 2 (9.52) | 3 (16.66) | 1 (20) | 1 (16.66) | 1 (20) | 0 | 0 | 2 (100) | 0 | 0 |
| 6 bachelor’s | 3 (14.28) | 2 (11.11) | 1 (20) | 1 (16.66) | 1 (20) | 0 | 0 | 0 | 0 | 1 (25) |
| 7 master’s | 0 | 0 | 0 | 0 | 0 | 0 | 0 | 0 | 0 | 0 |
| 8 doctoral | 0 | 0 | 0 | 0 | 0 | 0 | 0 | 0 | 0 | 0 |
| 9 other | 0 | 0 | 0 | 0 | 0 | 0 | 0 | 0 | 0 | 0 |
| **Surgery Type N (%)** | | | | | | | | | | |
| Coronary Bypass |  | 1 (5.55) |  |  |  |  |  | 1 (50)1 (50) |  |  |
| Cardiac valve replacement | 3 (14.28) | 1 (5.55) |  |  |  |  | 3 (100) |  |  |  |
| Scoliosis | 2 (9.52) | 4 (22.22) | 2 (33) | 4 (57) |  |  |  |  |  |  |
| Hip | 2 (9.52) | 2 (11.11) |  |  | 2 (33)4 (66) | 2 (50)2 (50) |  |  |  |  |
| Knee | 4 (19.05) | 2 (11.11) |  |  |  |  |  |  |  |  |
| Prostate | 2 (9.52) | 3 (16.66) |  |  |  |  |  |  | 2 (33) | 3 (60) |
| Kidney | 4 (19.05) | 2 (11.11) |  |  |  |  |  |  | 4 (66) | 2 (40) |
| Bladder | 4 (19.05) | 3(16.66) |  |  |  |  |  |  |  |  |
| Orthognathic |  |  | 4 (66) | 3 (42) |  |  |  |  |  |  |
| **Maxillofacial** | | | | | | | | | | |
| PAM-13 – baseline total raw score + per item separately: |  |  |  |  |  |  |  |  |  |  |
| mean±SD | 42.43±7.58 | 41.6±6.43 | 45 ±6.16 | 40.16±9.41 | 44.2±5.8 | 41.33±5.1 | 36.33±4.93 | 37 ±2.83 | 43±11.38 | 43.75±5.5 |
| Median (min-max) | 42 (23-52) | 42.5 (24-50) | 42 (38-52) | 43 (24-50) | 45 (36-50) | 40 (37-47) | 34 (33 - 42) | 37 (35-39) | 46 (23 - 51) | 43.5 (39 - 49) |
| **PAM-13 – Item 1** | | | | | | | | | | |
| mean±SD | 3.33 ±0.79 | 3.11±1.02 | 3.4±0.89 | 3±1.09 | 3.8±0.44 | 3±1.73 | 3±1 | 3.5±0.71 | 3±1 | 3.25±0.95 |
| Median (min-max) | 4(2 - 4) | 3 (1 - 4) | 4 (2 - 4) | 3 (1 - 4) | 4 (3 - 4) | 4(1-4) | 3 (2 - 4) | 3.5 (3 - 4) | 3 (2 - 4) | 3.5 (2 - 4) |
| PAM-13 – Item 2 |  |  |  |  |  |  |  |  |  |  |
| mean±SD | 3.24±0.89 | 3.39±0.98 | 2.8±1.95 | 3.16±1.17 | 3.6±0.54 | 3 ±1.73 | 3.33±0.57 | 3.5±0.71 | 3.2±1.3 | 3.75±0.5 |
| Median (min-max) | 3 (1 - 4) | 4 (1 - 4) | 3 (1 - 4) | 3.5 (1 - 4) | 4 (3 - 4) | 4(1-4) | 3 (3 - 4) | 3.5 (3 - 4) | 4 (1 - 4) | 4 (3 - 4) |
| **PAM-13 – Item 3** | | | | | | | | | | |
| mean±SD | 3.24±0.89 | 2.89±1.18 | 3.4±0.89 | 3±1.09 | 3.6±0.54 | 2.33 ±1.53 | 2.33±0.57 | 2±2.83 | 3.2±1.3 | 3.5±0.57 |
| Median (min-max) | 3 (1 - 4) | 3 (0 - 4) | 4 (2 - 4) | 3 (1 - 4) | 4 (3 - 4) | 2(1-4) | 2 (2- 3) | 2 (0 - 4) | 4 (1 - 4) | 3.5 (3 - 4) |
| **PAM-13 – Item 4** | | | | | | | | | | |
| mean±SD | 3.28±1.1 | 3.61±0.91 | 3.6±1.14 | 3.33±1.36 | 4±0 | 3.66±0.58 | 1.66±1.15 | 3.5±0.71 | 3.4±0.89 | 3.5±0.57 |
| Median (min-max) | 4 (1 - 5) | 4 (1 - 5) | 4 (2 - 5) | 3.5 (1 - 5) | 4 (4 - 4) | 4(3-4) | 1 (1 - 3) | 3.5 (3 - 4) | 4 (2- 4) | 3.5 (3 - 4) |
| **PAM-13 – Item 5** | | | | | | | | | | |
| mean±SD | 3.57±0.74 | 3.38±0.69 | 3.6±0.54 | 3.16±0.75 | 3.6±1.14 | 3.33 ±1.15 | 3.66±0.57 | 4±0 | 3.6±0.89 | 3.25±0.5 |
| Median (min-max) | 4 (2 - 5) | 3.5 (2 - 4) | 4 (3 - 4) | 3 (2 - 4) | 4 (2 - 5) | 4 (2 - 4) | 4 (3 - 4) | 4 (4 - 4) | 4 (2 - 4) | 3 (3 - 4) |
| **PAM-13 – Item 6** | | | | | | | | | | |
| mean±SD | 3.57±0.74 | 3.72±0.46 | 3.4±0.54 | 3.5±0.54 | 4±0.7 | 4±0 | 3.66±0.57 | 4±0 | 3.6±0.89 | 3.5±0.57 |
| Median (min-max) | 4 (2 - 5) | 4 (3- 4) | 3 (3 - 4) | 3.5 (3 - 4) | 4 (3 - 5) | 4 (4 - 4) | 4 (3 - 4) | 4 (4 - 4) | 4 (2 - 4) | 3.5 (3 - 4) |
| **PAM-13 – Item 7** | | | | | | | | | | |
| mean±SD | 3.57±0.59 | 3.72±0.46 | 3.8±0.44 | 3.66±0.51 | 3.6±0.54 | 4±0 | 3.66±0.57 | 4±0 | 3.6±0.89 | 3.5±0.57 |
| Median (min-max) | 4 (2 - 4) | 4 (3 - 4) | 4 (3 - 4) | 4 (3 - 4) | 4 (3 - 4) | 4 (4 - 4) | 3 (3 - 4) | 4 (4 - 4) | 4 (2 - 4) | 3.5(3- 4) |
| **PAM-13 – Item 8** | | | | | | | | | | |
| mean±SD | 3.33±1.01 | 3.22±0.87 | 3.8±0.83 | 2.5±1.05 | 4.2±0.44 | 4±0 | 2.66±0.57 | 3.5±0.71 | 3±1 | 3.5±0.57 |
| Median (min-max) | 3 (1 - 5) | 3(1 - 4) | 4 (3 - 5) | 2.5 (1 - 4) | 4 (4 - 5) | 4 (4 - 4) | 3 (2 - 3) | 3.5 (3 – 4) | 3 (2 - 4) | 3.5 (3 - 4) |
| **PAM-13 – Item 9** | | | | | | | | | | |
| mean±SD | 3.24±1.13 | 2.89±0.96 | 3.8±1.3 | 2.66±1.36 | 3.8±0.44 | 3.33 ±0.58 | 1.33±0.57 | 2±0 | 3.2±0.83 | 3.25±0.95 |
| Median (min-max) | 3 (1 - 5) | 3(1 - 4) | 4 (2 - 5) | 3 (1 - 4) | 4 (3 - 4) | 3 (3- 4) | 1 (1 - 2) | 2.5 (2 - 2) | 3 (2 - 4) | 3.5 (2 - 4) |
| **PAM-13 – Item 10** | | | | | | | | | | |
| mean±SD | 3.23±0.83 | 3.05±0.93 | 3.4±0.89 | 2.83±1.17 | 3.6±0.89 | 3.33 ±0.58 | 2.5±0.71 | 2.66±0.57 | 3.4±0.89 | 3±0.81 |
| Median (min-max) | 3 (2 - 4) | 3(1 - 5) | 4 (2 - 4) | 3 (1 - 4) | 4 (2 - 4) | 3 (3- 4) | 2.5 (2 - 3) | 3 (2 - 3) | 4 (2 - 4) | 3 (2 - 4) |
| PAM-13 – Item 11 |  |  |  |  |  |  |  |  |  |  |
| mean±SD | 3.28±0.78 | 3±0.84 | 3.4±1.14 | 3 ±1.26 | 3.6±0.54 | 3±1 | 3±0 | 3±0 | 3±1 | 3±0.81 |
| Median (min-max) | 3 (2 - 5) | 3 (1 - 4) | 3 (2 - 5) | 3.5 (1 - 4) | 4 (3 - 4) | 3 (2 - 4) | 3(3 - 3) | 3 (3 - 3) | 3 (2 - 4) | 3 (2 - 4) |
| **PAM-13 – Item 12** | | | | | | | | | | |
| mean±SD | 3.19±0.81 | 3.05±1.14 | 3.4±1.14 | 3.5±0.54 | 3.2±0.44 | 2.33±1.15 | 2.33±0.57 | 0 ±NaN | 3.4±0.89 | 3.75±0.95 |
| Median (min-max) | 3 (2 - 5) | 3 (0 - 5) | 3 (2 - 5) | 3.5 (3 - 4) | 3 (3 - 4) | 3 (1 - 3) | 2 (2 - 3) | 0 (0 - 0) | 4 (2 - 4) | 3.5 (3 - 5) |
| **PAM-13 – Item 13** | | | | | | | | | | |
| mean±SD | 3.28±0.9 | 3.11± 1.11 | 3.2±0.83 | 2.83±0.98 | 3.6 ±0.89 | 2±1 | 3.33±0.57 | 3±NaN | 3.4±1.34 | 4±0.81 |
| Median (min-max) | 3 (1 - 5) | 3 (1 - 5) | 3 (2 - 4) | 3 (1 - 4) | 3 (3 - 5) | 2 (1 - 3) | 3 (3 - 4) | 3 (3 - 3) | 4 (1 - 4) | 4 (3 - 5) |
| **EQ-5D baseline utility score** | | | | | | | | | | |
| Mean±SD | 7.65±2.39 | 6.5±2.17 | 7±3.74 | 5.5 ±0.83 | 9 ±0 | 9.33±4.04 | 9±2 | 7±1.41 | 6.2±1.64 | 5.5±0.57 |
| Median (min-max) | 8 (3-13) | 6 (5-14) | 7 (3-13) | 5 (5-7) | 9 (9-9) | 7 (7 - 14) | 9 (7 - 11) | 7 (6 - 8) | 5 (5 - 8) | 5.5 (5 - 6) |
| **EQ-5D baseline VAS score** | | | | | | | | | | |
| Mean±SD | 70.7±14.34 | 68 ±20.4 | 84 ±11.4 | 74.16±13.5 | 68±8.36 | 33.33±23.09 | 56.66±20.81 | 80±0 | 64±8.94 | 71.25±13.15 |
| Median (min-max) | 70 (40-100) | 72.5(20-90) | 80 (70-100) | 77.5(50-90) | 70 (60-80) | 20 (20 - 60) | 50 (40 – 80) | 80 (80 - 80) | 70(50 - 70) | 67.5 (60 -90) |
| **VAS pain – baseline** | | | | | | | | | | |
| Mean±SD | 31.9 ±33.1 | 29.4±37.64 | 16 ±35.77 | 23.3±30.1 | 64±8.94 | 26.66±46.18 | 0±0 | 0±0 | 10±12.25 | 10±8.16 |
| Median (min-max) | 10 (0-80) | 10 (0-100) | 0 (0-80) | 15 (0-80) | 70 (50-70) | 0 (0-80) | 0 (0 - 0) | 0 (0 - 0) | 10 (0 - 30) | 10 (0 - 20) |
| **VAS stress – baseline** | | | | | | | | | | |
| Mean±SD | 47.14±33 | 41.1±31.6 | 30 ±39.37 | 28.3±23.16 | 40 ±29.15 | 63.33±40.41 | 83.3±15.27 | 85± 7.07 | 40±35.35 | 32.5±17.08 |
| Median (min-max) | 60 (0-100) | 35 (0-100) | 10(0-90) | 25 (0-60) | 50 (0-70) | 70 (20 - 100) | 80 (70 - 100) | 85 (80 - 90) | 30 (0 - 90) | 35 (10 - 50) |
| **HADS – Depression - baseline** | | | | | | | | | | |
| Mean±SD | 5.28 ±5.8 | 5.88±5.31 | 0.8 ±0.83 | 4.5 ±4.93 | 3.4±2.5 | 3.66±2.08 | 4.33±1.53 | 8.5±0.71 | 11.8±8.64 | 10.75±7.8 |
| Median (min-max) | 4 (0-20) | 4 (0-18) | 1 (0-2) | 2.5 (0-12) | 2(1-7) | 3 (2 - 6) | 4 (3 - 6) | 8.5 (8 - 9) | 17 (1 - 20) | 10.5 (4 - 18) |
| **HADS – Anxiety - baseline** | | | | | | | | | | |
| Mean±SD | 7.95 ±6.12 | 9.3±5.1 | 4.6 ±8.1 | 10±4.81 | 6.2 ±4 | 9.33±3.05 | 9.66±2.08 | 6.5±6.36 | 11.6±8.02 | 12±7.5 |
| Median (min-max) | 8 (0-19) | 8.5 (2-19) | 1(0-19) | 8.5 (6-19) | 7 (2-10) | 10 (6 - 12) | 9 (8 - 12) | 6.5 (2 - 11) | 12 (0 -19) | 12 (5 - 19) |
| **PANAS – Positive - baseline** | | | | | | | | | | |
| Mean±SD | 29.71±7 | 27.8 ±7.53 | 27.8 ±5.76 | 30.16 ±9.2 | 33±5 | 30.33±12.01 | 32.33±3.21 | 20±0 | 29.2±7.46 | 27.75±2.63 |
| Median (min-max) | 31 (16-41) | 28 (16-44) | 31 (19-33) | 30.5 (16-44) | 34 (25-38) | 31 (18 - 42) | 31 (20 - 36) | 20 (20 - 20) | 29(20 - 38) | 28.5(24 - 30) |
| **PANAS – Negative - baseline** | | | | | | | | | | |
| Mean±SD | 19.38 ±9.38 | 18 ±6.63 | 18 ±13.17 | 17.83±6.3 | 16 ±4.53 | 21±8.18 | 23±6 | 25±5.65 | 21.8±12.61 | 16±7.61 |
| Median (min-max) | 17(10-41) | 15.5(10-29) | 12 (10-41) | 15.5 (12-28) | 17 (11-22) | 23 (12 - 28) | 23 (17 - 29) | 25 (21 - 29) | 17(11 -39 ) | 13.5 (10 - 27) |
| **SWEMWBS – baseline (raw score)** | | | | | | | | | | |
| Mean±SD | 25.38±4.62 | 25.27±4.76 | 23.6 ±5.17 | 26±5.5 | 29.47±3.32 | 24.66±3.78 | 27.66±1.52 | 18±0 | 24.4±5.32 | 26±2.58 |
| Median (min-max) | 26(17-33) | 24.5(18-33) | 22 (18-30) | 25.5 (18-33) | 25.03(23.2-30.7) | 23 (22 - 29) | 28(26 - 29) | 18 (18 - 18) | 24 (17- 30) | 26 (23 - 29) |
| **SWEMWBS – baseline (metric sc)** | | | | | | | | | | |
| Mean±SD | 23.68±3.75 | 23.04±4.13 | 22.36±4.43 | 23.8±4.97 | 26.47±3.32 | 22.24±3.29 | 24.75±1.42 | 17.4±0 | 22.3±4.24 | 23.3±2.27 |
| Median (min-max) | 24.1(16.8-30.7) | 21.9(17.4- 30.7) | 22.5 (17.4- 27.0) | 22.8(17.4 – 30.7) | 25.03(23.2-30.7) | 20.7(19.9 – 26.0) | 25 (23.21-26) | 17.4(17.4 – 17.4) | 21.5(16.8 – 27.0) | 23.2(20.7 - 26.0) |
| **GSE - baseline** | | | | | | | | | | |
| Mean±SD | 30.66±6.03 | 27.83 ±9.74 | 28.8 ±5.16 | 30.16±6.91 | 32.6±7.83 | 29.33±9.61 | 29±2.64 | 6±8.48 | 32.4±7.86 | 31.25±2.21 |
| Median (min-max) | 32 (19-39) | 30.5 (0-38) | 32(21-33) | 31.5(19-37) | 36(19-38) | 31 (19 - 38) | 30(26- 31) | 6 (0 - 12) | 34(19 - 39) | 31(29-34) |
| **Medical history#** | | | | | | | | | | |
| Active cancer N (%) | 6 (28.57) | 5 (27.77) | 0 (0) | 0 (0) | 0 (0) | 0 (0) | 0 (0) | 0 (0) | 5 (100) | 4 (100) |
| Cardiovascular disease N (%) | 3 (14.28) | 4 (22.22) | 0 (0) | 0 (0) | 0 (0) | 1 (33.33) | 3 (100) | 2(100) | 0 (0) | 0 (0) |
| Cerebrovascular disease N (%) | 0 (0) | 0 (0) | 0 (0) | 0 (0) | 0 (0) | 0 (0) | 0 (0) | 0 (0) | 0 (0) | 0 (0) |
| Diabetes Mellitus N (%) | 1 (4.76) | 0 (0) | 0 (0) | 0 (0) | 1 (33.33) | 0 (0) | 0 (0) | 0 (0) | 0 (0) | 0 (0) |
| Pulmonary disease N (%) | 0 (0) | 0 (0) | 0 (0) | 0 (0) | 0 (0) | 0 (0) | 0 (0) | 0 (0) | 0 (0) | 0 (0) |
| Renal disease N (%) | 1 (4.76) | 0 (0) | 0 (0) | 0 (0) | 1 (33.33) | 0 (0) | 0 (0) | 0 (0) | 0 (0) | 0 (0) |
| Anxiety disorder N (%) | 2 (9.52) | 0 (0) | 0 (0) | 0 (0) | 1 (33.33) | 0 (0) | 0 (0) | 0 (0) | 0 (0) | 0 (0) |
| Depression N (%) | 1 (4.76) | 0 (0) | 0 (0) | 0 (0) | 0 (0) | 0 (0) | 0 (0) | 0 (0) | 0 (0) | 0 (0) |
| Cognitive disorder N (%) | 1 (4.76) | 0 (0) | 1 () | 0 (0) | 0 (0) | 0 (0) | 0 (0) | 0 (0) | 0 (0) | 0 (0) |
| **Medication Use#** | | | | | | | | | | |
| Sleep medication N (%) | 1 (4.76) | 1 (5.55) | 0 (0) | 1 (16.66) | 1 (20) | 0 (0) | 0 (0) | 0 (0) | 0 (0) | 0 (0) |
| Pain medication N (%) | 5 (23.8) | 5 (27.77) | 0 (0) | 2 (33.33) | 4 (80) | 2 (66.66) | 0 (0) | 1 (50) | 0 (0) | 0 (0) |
| Anxiety medication N (%) | 3 (14.28) | 1 (5.55) | 0 (0) | 1 (16.66) | 2 (40) | 0 (0) | 0 (0) | 0 (0) | 0 (0) | 0 (0) |
| **Digital expertise#** | | | | | | | | | | |
| 1. none N (%) | 1 (4.76) | 0 (0) | 0 (0) | 0 (0) | 1 (20) | 0 (0) | 0 (0) | 0 (0) | 0 (0) | 0 (0) |
| 2. Basic skills N (%) | 10 (47.62) | 3 (16.66) | 1 (20) | 0 (0) | 3 (60) | 2 (66.66) | 3 (100) | 0 (0) | 2 (40) | 1 (25) |
| 3. Advanced N (%) | 6 (28.57) | 11 (61.11) | 1 (20) | 3 (50) | 1 (20) | 1 (33.33) | 0 (0) | 1 (50) | 3 (60) | 3 (75) |
| 4. Expert N (%) | 4 (19.05) | 4 (22.22) | 3 (60) | 3 (50) | 0 (0) | 0 (0) | 0 (0) | 1 (50) | 0 (0) | 0 (0) |
| **Illiteracy**# | | | | | | | | | | |
| 1. Analphabetic   Low skills N (%) |  |  |  |  |  |  |  |  |  |  |
| Basic skills N (%) | 4 (19.04) | 1 (5.88) | 1 (20) |  |  |  |  |  | 2 (40) | 1 (25) |
| Fluent N (%) | 17 (80.95) | 16 (94.11) | 4 (80) | 6 (100) | 5 (100) | 5 (100) | 3 (100) | 2 (100) | 3 (60) | 3 (75) |
| **Time included in the study** (in days)# | | | | | | | | | | |
| Mean±SD | 33.9±18 | 21.2 ±16.05 | 21.2±16.05 | 24.33±5.31 | 27.4±12.72 | 37±28.93 | 33±2.64 | 27.5±3.53 | 54.4±16.92 | 31.75±4.35 |
| Median (min-max) | 35 (9 - 71) | 14 (9 - 48) | 14 (9 - 48) | 23 (19 - 33) | 36 (13 - 38) | 25 (16 - 70) | 34 (30 - 35) | 27.5 (25 - 30) | 62 (28 - 71) | 31.5 (28 - 36) |
| **Caregiver** | | | | | | | | | | |
| **N** | 7 | 10 | 3 | 6 | 0 | 2 | 3 | 2 | 1 | 0 |
| **VAS stress – baseline** | | | | | | | | | | |
| Mean±SD | 40±37.86 | 69 ±32.13 | 23.33±40.41 | 66.66±20.65 |  | 50±70.71 | 70±10 | 95±7.07 | 0±NaN |  |
| Median (min-max) | 60 (0-80) | 70 (0-100) | 0 (0-70) | 70 (40-100) |  | 50 (0 - 100) | 70 (90 - 80) | 95 (90 - 100) | 0 (0 - 0) |  |
| **SWEMWBS – baseline(raw)** | | | | | | | | | | |
| Mean±SD | 28.43 ±3.64 | 21.8±9.7 | 31.6 ±1.52 | 21.6 ±7.55 |  | 18.33±16.8 | 27±2 | 27.5±2.12 | 23±NaN |  |
| Median (min-max) | 29(23-33) | 24 (0-33) | 32 (30-33) | 24 (8-30) |  | 22 (0 - 33) | 27 (25-29) | 27.5 (26 - 29) | 23 (23 -23) |  |
| **SWEMWBS – baseline(metric)** | | | | | | | | | | |
| Mean±SD | 25.75±3.61 | 22.16 ±5.32 | 29.01±1.85 | 20.28 ±5.28 |  | 25.34±7.58 | 24.16±1.83 | 24.61±1.98 | 20.73±NaN |  |
| Median (min-max) | 26 (20.73- 30.7) | 21.9(11.2 – 30.7) | 29.3(27.03-30.7) | 21.5(11.2-27.0) |  | 25.3(19.9 – 30.7) | 24.1 (22.3-26.0) | 24.6(23.2- 26.0) | 20.7 (20.7 -20.7) |  |
| **GSE – baseline** | | | | | | | | | | |
| Mean±SD | 29.29±3.0 | 24.72±9.62 | 34.33±2.39 | 25.33±3.54 |  | 19.67±14.38 | 26±2.64 | 30.5± 9.19 | 24±0 |  |
| Median (min-max) | 29.2(24.0 - 37.0) | 25 (0-37) | 34.3(30.0 - 37.0) | 25.3(20.0 - 29.0) |  | 22.33(0.0 - 34.0) | 25(24-29) | 30.5 (24 - 37) | 24 (24 -24) |  |

# NaN = Not Available
