# Supplementary material for: Stress Reduction in Perioperative Care: Feasibility Randomized Controlled Trial
Source: J Med Internet Res. 2025 Jan 7;27:e54049. doi: 10.2196/54049 (PMC11751654; doi:10.2196/54049)
Supplement: Multimedia Appendix 3 [file jmir_v27i1e54049_app3.docx]

| **Patients** | **Overall** | | | **Hospital HSJD** | | | **Hospital Parc Taulí** | | | **Hospital SAS** | | | **Hospital INRCA** | | |
| --- | --- | --- | --- | --- | --- | --- | --- | --- | --- | --- | --- | --- | --- | --- | --- |
|  | Control | Intervention | p-value* | Control | Intervention | p-value* | Control | Intervention | p-value* | Control | Intervention | p-value* | Control | Intervention | p-value* |
| **VAS pain – hospital admission** | 30.4±31.5 | 20.9±28.5 | 0.33 | 10±10 | 21.6±34.8 | 0.92 | 62 ±8.36 | 45.6±41.7 | 0.40 | 26.6±37.8 | 40±26.4 | 0.67 | 6±13.41 | 15±12.9 | 0.28 |
| Mean±SD  Median (min-max) | 20 (0-90) | 8.5 (0-90) |  | 10 (0-20) | 0 (0-80) |  | 60 (50-70) | 40 (7-90) |  | 10 (0 - 70) | 50 (10-60) |  | 0 (0-30) | 15 (0-30) |  |
| **VAS pain – hospital discharge**  Mean±SD | 51.3± 33.8 | 51.1±30.6 | 0.98 | 44±37.8 | 56.6±30.7 | 0.55 | 63.3±8.1 65 | 56.6±32.1 | 0.47 | 66.6±20.8 | 40±26.4 | 0.36 | 16±16.7 | 37.5±30.9 | 0.22 |
| Median (min-max) | 60 (0-100) | 65 (0-90) |  | 40 (10-100) | 65 (0-90) |  | (50-70) | 70 (20-80) |  | 60 (50-90) | 50 (10-60) |  | 20 (0-40) | 30 (10-80) |  |
| **VAS pain – POD14**  Mean±SD | 35±23.0 | 37.7±26.6 | 0.71 | 22±27.7 | 38.3±27.8 | 0.35 | 49±15.9 | 56.6±23.0 | 0.64 | 46.6±11.5 | 90±17.3 | 0.10 | 22±22.8 | 17.5±14.43 | 0.74 |
| Median (min-max) | 40 (0-70) | 32.5 (0-70) |  | 10 (0-70) | 40 (10-70) |  | 45 (30-70) | 70 (30-70) |  | 40 (40-60) | 50 (10-60) |  | 20 (0-60) | 17.5 (0-35) |  |
| **VAS stress – hosp admission**  Mean±SD | 54.2±32.1 | 51.3±32.8 | 0.87 | 32±25.8 | 30±26.07 | 0.9 | 56±32.0 | 75±27.8 | 0.45 | 90±17.32 | 40±26.4 | 0.17 | 40±33.1 | 45±26.45 | 0.81 |
| Median (min-max) | 70 (0-100) | 47.5 (0-100) |  | 30 (0-70) | 25 (0-70) |  | 70 (0-80) | 80 (45-100) |  | 100 (70-100) | 50 (10-60) |  | 50 (0-70) | 40 (20-80) |  |
| **VAS stress – hosp discharge**  Mean±SD | 39.4±34.55 | 35±25.26 | 0.85 | 38± 35.6 | 38.3±20.4 | 0.85 | 30±30.8 | 16.6±28.8 | 0.63 | 10 ± 10 | 40±26.45 | 0.33 | 28±26.6 | 35±33.16 | 0.77 |
| Median (min-max) | 40 (0-90) | 45 (0-80) |  | 40 (0-90) | 50 (0-50) |  | 30 (0-70) | 0 (0-50) |  | 10 (0-20) | 50 (10-60) |  | 10 (0-80) | 25 (10-80) |  |
| **VAS stress – POD14**  Mean±SD | 22.8±22.1 | 18.6±22.3 | 0.53 | 14±20.7 | 17.5±26.0 | 0.92 | 30±28.2 | 23.3±40.4 | 1 | 16.6± 20.8 | 40±26.4 | 0.41 | 20±21.2 | 22.5±15 | 0.73 |
| Median (min-max) | 20 (0-60) | 10 (0-70) |  | 10 (0-50) | 2.5 (0-60) |  | 40 (0-60) | 0 (0-70) |  | 10 (0-40) | 50 (10-60) |  | 20 (0-50) | 20 (10-40) |  |
| **HADS – Anxiety – hospital adm** | 6.55 ±3.38 | 6.7±4.5 | 0.85 | 6.2±4.6 | 5.8±4.1 | 0.89 | 6±2.5 | 11±6.08 | 0.14 | 6.33±1.15 | 40±26.45 | 0.32 | 6 ±5.16 | 5 ±2.58 | 0.70 |
| Mean±SD  Median (min-max) | 7 (0-13) | 6 (0-15) |  | 6 (2-13) | 5.5 (1-12) |  | 6 (3-9) | 14 (4-15) |  | 7 (5-7) | 50 (10-60) |  | 6 (0-12) | 5 (2-8) |  |
| **HADS – Anxiety – hospital disch**  Mean±SD | 5.3±3.54 | 5.27±3.8 | 0.97 | 5.6±4.16 | 6.6±4.67 | 0.7 | 4±2.74 | 2.33±4.04 | 0.64 | 5±2.64 | 40±26.45 | 0.31 | 3.6±3.21 | 5.25±2.98 | 0.45 |
| Median (min-max) | 5 (0-12) | 6 (0-14) |  | 4 (2-11) | 6.5 (1-14) |  | 4 (0-7) | 0 (0-7) |  | 6 (2-7) | 50 (10-60) |  | 4 (0-8) | 6 (1-8) |  |
| **HADS – Anxiety – POD14**  Mean±SD | 5±3.46 | 4.22±3.19 | 0.47 | 5±4.06 | 6±4.2 | 0.7 | 3±2.23 | 2.66±3.05 | 0.86 | 6.33± 3.51 | 40±26.45 | 0.32 | 4.4±4.16 | 4.25±2.22 | 0.95 |
| Median (min-max) | 4 (0-11) | 3.5 (0-12) |  | 4 (1-11) | 5 (1-12) |  | 3 (0-6) | 2 (0-6) |  | 6 (3-10) | 50 (10-60) |  | 4 (0-10) | 4 (2-7) |  |
| **HADS – Depression – hospital adm**  Mean±SD | 5.2±4.86 | 5.16±4.59 | 0.98 | 1.8±2.49 | 3.66±4.67 | 0.77 | 2±1.41 | 7.66±3.78 | 0.03* | 11.3±5.89 | 40±26.45 | 0.36 | 8±5.35 | 3.75±2.75 | 0.15 |
| Median (min-max) | 4 (0-18) | 4 (0-15) |  | 1 (0-6) | 1.5 (0-10) |  | 3 (0-3) | 6 (5-12) |  | 9 (7-18) | 50 (10-60) |  | 7.5 (2-15) | 3.5 (1-7) |  |
| **HADS – Depr – hospital disch**  Mean±SD | 4.84±4.37 | 5.33±4.13 | 0.69 | 6±7.58 | 6.16±4.66 | 0.96 | 2.2±2.39 | 1.66 ±1.53 | 0.744 | 5.33±4.04 | 40±26.45 | 0.31 | 4.6±1.67 | 5.5±5.44 | 0.73 |
| Median (min-max) | 4 (0-19) | 4.5 (0-14) |  | 3 (0-19) | 5.5 (0-14) |  | 1 (0-6) | 2 (0-3) |  | 3 (3-10) | 50 (10-60) |  | 5 (3-7) | 4 (1-13) |  |
| **HADS – Depression – POD14**  Mean±SD |  |  | 0.85 |  |  | 0.58 |  |  | 0.34 |  |  | 0.31 |  |  | 0.25 |
|  | 4.57±3.12 | 4.44±3.73 |  | 4±4.18 | 5.5±4.5 |  | 1.6±1.14 | 4±5.29 |  | 6±3.46 | 40±26.45 |  | 5.2±1.09 | 3.25±4.5 |  |
| Median (min-max) | 4 (0-9) | 3.5 (0-11) |  | 2 (0-9) | 5 (0-11) |  | 2 (0-3) | 2 (0-10) |  | 8 (2 -8) | 50 (10-60) |  | 6 (4-6) | 1 (1-10) |  |
| **Length of hospital stay (days)**  Mean±SD | 5.43±7.75 | 4.5±7.4 | 0.6 | 1.8± 2.38 | 1.66±1.21 | 0.7 | 4.6±5.81 | 4±3.46 | 1 | 8.66± 3.21 | 40±26.45 | 0.34 | 9.6±14.2 | 3±1.15 | 0.51 |
| Median (min-max) | 2 (0-35) | 2 (0 - 33) |  | 1 (0 - 6) | 1.5 (0 - 3) |  | 2 (2 - 15) | 2 (2 - 8) |  | 10 (5-11) | 50 (10-60) |  | 4 (2-35) | 3 (2 - 4) |  |
| **Destination after discharge** |  |  |  |  |  |  |  |  |  |  |  |  |  |  |  |
| Home N (%) |  |  |  |  |  |  |  |  |  |  |  |  |  |  |  |
| Nursing home N (%) | 21 (100) | 18 (100) |  | 6 (50) | 7 (50) |  | 6 (50) | 4 (50) |  | 2 (50) | 3 (50) |  | 6 (50) | 5 (50) |  |
| Rehabilitation centre N (%) |  |  |  |  |  |  |  |  |  |  |  |  |  |  |  |
| Other hospital N (%) |  |  |  |  |  |  |  |  |  |  |  |  |  |  |  |
| Other N (%) |  |  |  |  |  |  |  |  |  |  |  |  |  |  |  |
| **Medication use POD14#** |  |  |  |  |  |  |  |  |  |  |  |  |  |  |  |
| Pain medication N (%) | 1(4.76) | 8(44.4) |  | 5 (100) | 5 (83.3) |  | 2 (40) | 2 (66.6) |  | 0 (0) | 0 (0) |  | 0 (0) | 0 (0) |  |
| Stress medication N (%) | 1(4.76) | 1 (5.55) |  | 0 (0) | 1 (16.6) |  | 1 (20) | 0 (0) |  | 0 (0) | 0 (0) |  | 0 (0) | 0 (0) |  |
| Sleep medication N (%) | 0 (0) | 1 (5.55) |  | 0 (0) | 1 (16.6) |  | 0 (0) | 0 (0) |  | 0 (0) | 0 (0) |  | 0 (0) | 0 (0) |  |
| **Postop complications#** | | | | | | | | | | | | | | | |
| Any N (%) | 1 (4.76) | 1 (5.55) |  | 0 (0) | 1 (5.55) |  | 1 (4.76) | 0 (0) |  | 0 (0) | 0 (0) |  | 0 (0) | 0 (0) |  |
| Per category: |  |  |  |  |  |  |  |  |  |  |  |  |  |  |  |
| Infectious N (%) | 0 (0) | 0 (0) |  | 0 (0) | 0 (0) |  | 0 (0) | 0 (0) |  | 0 (0) | 0 (0) |  | 0 (0) | 0 (0) |  |
| Cardiac N (%) | 0 (0) | 0 (0) |  | 0 (0) | 0 (0) |  | 0 (0) | 0 (0) |  | 0 (0) | 0 (0) |  | 0 (0) | 0 (0) |  |
| Pulmonary N (%) | 0 (0) | 0 (0) |  | 0 (0) | 0 (0) |  | 0 (0) | 0 (0) |  | 0 (0) | 0 (0) |  | 0 (0) | 0 (0) |  |
| Vascular N (%) | 0 (0) | 1 (5.55) |  | 0 (0) | 1 (5.55) |  | 0 (0) | 0 (0) |  | 0 (0) | 0 (0) |  | 0 (0) | 0 (0) |  |
| Renal N (%) | 0 (0) | 1 (5.55) |  | 0 (0) | 1 (5.55) |  | 0 (0) | 0 (0) |  | 0 (0) | 0 (0) |  | 0 (0) | 0 (0) |  |
| Surgical N (%) | 0 (0) | 1 (5.55) |  | 0 (0) | 1 (5.55) |  | 0 (0) | 0 (0) |  | 0 (0) | 0 (0) |  | 0 (0) | 0 (0) |  |
| Delirium N (%) | 0 (0) | 1 (5.55) |  | 0 (0) | 1 (5.55) |  | 0 (0) | 0 (0) |  | 0 (0) | 0 (0) |  | 0 (0) | 0 (0) |  |
| Other N (%) | 1 (4.76) | 1 (5.55) |  | 0 (0) | 1 (5.55) |  | 1 (4.76) | 0 (0) |  | 0 (0) | 0 (0) |  | 0 (0) | 0 (0) |  |
| **Highest complication grade (Clavien-Dindo classification)#** | | | | | | | | | | | | | | | |
|  |  |  |  |  |  |  |  |  |  |  |  |  |  |  |  |
| I N (%) | 0 (0) | 0 (0) |  | 0 (0) | 0 (0) |  | 0 (0) | 0 (0) |  | 0 (0) | 0 (0) |  | 0 (0) | 0 (0) |  |
| II N (%) | 1 (4.76) | 0 (0) |  | 0 (0) | 0 (0) |  | 1 (4.76) | 0 (0) |  | 0 (0) | 0 (0) |  | 0 (0) | 0 (0) |  |
| III N (%) | 0 (0) | 1 (5.55) |  | 0 (0) | 1 (5.55) |  | 0 (0) | 0 (0) |  | 0 (0) | 0 (0) |  | 0 (0) | 0 (0) |  |
| III-a N (%) | 0 (0) | 1 (5.55) |  | 0 (0) | 1 (5.55) |  | 0 (0) | 0 (0) |  | 0 (0) | 0 (0) |  | 0 (0) | 0 (0) |  |
| III-b N (%) | 0 (0) | 0 (0) |  | 0 (0) | 0 (0) |  | 0 (0) | 0 (0) |  | 0 (0) | 0 (0) |  | 0 (0) | 0 (0) |  |
| IV N (%) | 0 (0) | 0 (0) |  | 0 (0) | 0 (0) |  | 0 (0) | 0 (0) |  | 0 (0) | 0 (0) |  | 0 (0) | 0 (0) |  |
| IV-a N (%) | 0 (0) | 0 (0) |  | 0 (0) | 0 (0) |  | 0 (0) | 0 (0) |  | 0 (0) | 0 (0) |  | 0 (0) | 0 (0) |  |
| IV-b N (%) | 0 (0) | 0 (0) |  | 0 (0) | 0 (0) |  | 0 (0) | 0 (0) |  | 0 (0) | 0 (0) |  | 0 (0) | 0 (0) |  |
| V N (%) | 0 (0) | 0 (0) |  | 0 (0) | 0 (0) |  | 0 (0) | 0 (0) |  | 0 (0) | 0 (0) |  | 0 (0) | 0 (0) |  |
| **Recovery VAS#**  Mean±SD | 62.3±21.1 | 60.4±31.3 | 0.91 | 70 ±15.8 | 55.1±33.5 | 0.39 | 55±5.77 | 40±26.45 | 0.24 | 50± 10 | 40±26.45 | NaN | 69.4±37.9 | 95 ±5.77 | 0.36 |
| Median (min-max) | 60 (7-100) | 60 (7-100) |  | 70 (50-90) | 65 (14-99) |  | 55 (50-60) | 50 (10-60) |  | 50 (40-60) | 50 (10-60) |  | 70 (7-100) | 95 (90-100) |  |
| **Wound healing#** |  |  | NaN |  |  | NaN |  |  | NaN |  |  | NaN |  |  | Na |
| Days to removal of stitches |  |  |  |  |  |  |  |  |  |  |  |  |  |  | N |
| Mean±SD | 0±NaN | 2±NaN |  | 0±NaN | 2±NaN |  | 0±NaN | 0±NaN |  | 0±NaN | 0±NaN |  | 0±NaN | 0±NaN |  |
| Median (min-max) | 0(0-0) | 2(2-2) |  | 0(0-0) | 2(2-2) |  | 0(0-0) | 0(0-0) |  | 0(0-0) | 0(0-0) |  | 0(0-0) | 0(0-0) |  |
| **Re-hospitalization (<POD14)** | 0 (0) | 0 (0) |  | 0 (0) | 0 (0) |  | 0 (0) | 0 (0) |  | 0 (0) | 0 (0) |  | 0 (0) | 0 (0) |  |
| N (%) | 0 (0) | 0 (0) |  | 0 (0) | 0 (0) |  | 0 (0) | 0 (0) |  | 0 (0) | 0 (0) |  | 0 (0) | 0 (0) |  |
| **Re-operations (<POD14)** |  |  |  |  |  |  |  |  |  |  |  |  |  |  |  |
| N (%) | 0 (0) | 0 (0) |  | 0 (0) | 0 (0) |  | 0 (0) | 0 (0) |  | 0 (0) | 0 (0) |  | 0 (0) | 0 (0) |  |
| **Mortality (<POD14)** |  |  |  |  |  |  |  |  |  |  |  |  |  |  |  |
| N (%) | 0 (0) | 1 (1) |  | 0 (0) | 0 (0) |  | 0 (0) | 0 (0) |  | 0 (0) | 1(1) |  | 0 (0) | 0 (0) |  |

POD: PostOperative Day

# NaN = Not Available
