# Supplementary material for: Stress Reduction in Perioperative Care: Feasibility Randomized Controlled Trial
Source: J Med Internet Res. 2025 Jan 7;27:e54049. doi: 10.2196/54049 (PMC11751654; doi:10.2196/54049)
Supplement: Multimedia Appendix 4 [file jmir_v27i1e54049_app4.docx]

| **Patients** | **Overall** | | | | **Hospital HSJD** | | | **Hospital Parc Taulí** | | | **Hospital SAS** | | | **Hospital INRCA** | | |
| --- | --- | --- | --- | --- | --- | --- | --- | --- | --- | --- | --- | --- | --- | --- | --- | --- |
|  | Control | | Intervention | p-value* | Control | Intervention | p-value* | Control | Intervention | p-value* | Control | Intervention | p-value* | Control | Intervention | p-value* |
| **PAM-13 – hospital admission** total raw score:  mean±SD | 42.4±7.1 | | 45.7± 4.18 | 0.12 | 45.8±5.0 | 44.1±4.1 | 0.57 | 44.6± 6.3 | 49.6± 0.5 | 0.23 | 33.6± 6.4 | 49.6± 0.5 | 0.04 | 44.2±7.6 | 46.5±4.4 | 0.71 |
| Median (min-max) | 45 (29-54) | | 46 (39-51) |  | 45 (40-54) | 44.5 (39-50) |  | 47 (37-52) | 50 (49-50) |  | 31 (29-41) | 50 (49-50) |  | 48 (31-49) | 47 (41-51) |  |
| **PAM-13 – hospital admission** item1:  mean±SD | 3.2±0.7 | | 3.5±0.62 | 0.41 | 3.8±0.45 | 3.5± 0.55 | 0.38 | 3.4±0.54 | 3.6±0.57 | 0.61 | 2.66±0.5 | 3.6±0.57 | 0.15 | 3±1.2 | 3.75±0.5 | 0.34 |
| Median (min-max) | 3 (1-4) | | 4 (2-4) |  | 4 (3-4) | 4 (3-4) |  | 3 (3-4) | 4 (3-4) |  | 3 (2-3) | 4 (3-4) |  | 3 (1-4) | 4 (3-4) |  |
| **PAM-13 – hospital admission** item2: | 3.24± 0.7 | | 3.6±0.5 | 0.11 | 3.4±0.55 | 3.5±0.55 | 0.83 | 3.4±0.55 | 4±0 | 0.16 | 3±1 | 4 ±0 | 0.22 | 3±1.22 | 3.75±0.5 | 0.34 |
| mean±SD  Median (min-max) | 3 (1-4) | | 4 (3-4) |  | 3 (3-4) | 3.5 (3-4) |  | 3 (3-4) | 4 (4-4) |  | 3 (2-4) | 4 (4-4) |  | 3 (1-4) | 4 (3-4) |  |
| **PAM-13 – hospital admission** item3:  mean±SD | 3.33±0.9 | | 3.55 ±0.61 | 0.53 | 3.8±1.09 | 3.3±0.96 | 0.44 | 3.4±0.55 | 4± 0 | 0.16 | 2.6±1.15 | 4±0 | 0.18 | 3.4±1.34 | 3.5±0.57 | 0.66 |
| Median (min-max) | 4 (1-5) | | 4 (2-4) |  | 4 (2-5) | 4 (1-5) |  | 3 (3-4) | 4 (4-4) |  | 2 (2-4) | 4 (4-4) |  | 4 (1-4) | 3.5 (3-4) |  |
| **PAM-13 – hospital admission** item4:  mean±SD | 3.14±1.2 | | 3.55±0.78 | 0.33 | 3.6±1.51 | 3.5±0.78 | 0.55 | 3.6±0.54 | 4±0 | 0.32 | 1.66±1.1 | 4±0 | 0.07 | 3.2±1.3 | 4±0 | 0.66 |
| Median (min-max) | 3 (1-5) | | 4 (2-5) |  | 4 (2-5) | 4 (2-5) |  | 4 (3-4) | 4 (4-4) |  | 1 (1-3) | 4 (4-4) |  | 4 (1-4) | 4 (4-4) |  |
| **PAM-13 – hospital admission** item5:  mean±SD | 3.1±0.8 | | 3.61±0.5 | 0.07 | 3±0.7 | 3.5±0.55 | 0.26 | 3.6±0.55 | 4±0 | 0.32 | 3±1 | 4±0 | 0.22 | 3.2±1.3 | 3.25±0.5 | 0.68 |
| Median (min-max) | 5 3 (1-4) | | 4 (3-4) |  | 3 (2-4) | 3.5 (3-4) |  | 4 (3-4) | 4 (4-4) |  | 3 (2-4) | 4 (4-4) |  | 4 (1-4) | 3 (3-4) |  |
| **PAM-13 – hospital admission** item6:  mean±SD | 3.5±0.59 | | 3.66±0.48 | 0.69 | 3.4±0.55 | 3.5±0.55 | 0.83 | 3.6±0.55 | 4±0 | 0.32 | 4±0 | 4±0 | NaN | 3.8±0.44 | 3.5±0.57 | 0.45 |
| Median (min-max) | 4 (2-4) | | 4 (3-4) |  | 3 (3-4) | 3.5 (3-4) |  | 4 (3-4) | 4 (4-4) |  | 4 (4-4) | 4 (4-4) |  | 4 (3-4) | 3.5 (3-4) |  |
| **PAM-13 – hospital admission** item7:  mean±SD | 3.52±0.6 | | 3.77±0.55 | 0.23 | 3.8 ±0.44 | 3.5±0.55 | 0.38 | 3.4±0.54 | 4±0 | 0.16 | 3 ±1 | 4±0 | 0.22 | 3 .8±0.44 | 3.5±0.57 | 0.45 |
| Median (min-max) | 4 (2-4) | | 4 (3-5) |  | 4 (3-4) | 3.5 (3-4) |  | 3 (3-4) | 4 (4-4) |  | 3 (2-4) | 4 (4-4) |  | 4 (3-4) | 3.5 (3-4) |  |
| **PAM-13 – hospital admission** item8:  mean±SD | 3.35±0.9 | | 3.61±0.69 | 0.5 | 4.2±0.45 | 3.66±1.0 | 0.39 | 3.4±0.54 | 4±0 | 0.15 | 2±0 | 4 ±0 | 0.00 | 3.75±0.5 | 3.5±0.57 | 0.68 |
| Median (min-max) | 4 (1-5) | | 4 (2-5) |  | 4 (4-5) | 4 (2-5) |  | 3 (3-4) | 4 (4-4) |  | 2 (2-2) | 4 (4-4) |  | 4 (3-4) | 3.5 (3-4) |  |
| **PAM-13 – hospital admission** item9:  mean±SD | 3.04±1.0 | | 3.5± 0.7 | 0.11 | 3.4±1.14 | 3.3±0.81 | 0.91 | 3.2±0.45 | 3.6±0.57 | 0.29 | 1.3±0.57 | 3.6±0.57 | 0.02 | 3.4±0.89 | 4±0 | 0.24 |
| Median (min-max) | 3 (1-5) | | 4 (2-4) |  | 3 (2-5) | 3.5 (2-4) |  | 3 (3-4) | 4 (3-4) |  | 1 (1-2) | 4 (3-4) |  | 4 (2-4) | 4 (4-4) |  |
| **PAM-13 – hospital admission** item10:  mean±SD | 3.1±0.8 | | 3.22±0.87 | 0.9 | 3.4±0.89 | 3±0.63 | 0.37 | 3.4±0.89 | 3.6±0.57 | 0.86 | 2±1 | 3.66±0.5 | 0.74 | 3.6±0.55 | 3.5±0.57 | 0.88 |
| Median (min-max) | 3 (1-4) | | 3 (1-4) |  | 4 (2-4) | 3 (2-4) |  | 4 (2-4) | 4 (3-4) |  | 2 (1-3) | 4 (3-4) |  | 4 (3-4) | 3.5 (3-4) |  |
| **PAM-13 – hospital admission** item11:  mean±SD | 3.2±0.77 | | 3.27±0.57 | 0.81 | 3.2±1.09 | 3.16±0.4 | 0.81 | 3.6±0.54 | 3.3±1.15 | 1 | 2.6±0.57 | 3.3±1.15 | 0.55 | 3.4±0.89 | 3.5±0.57 | 1 |
| Median (min-max) | 3 (2-5) | | 2 (2-4) |  | 3 (2-5) | 3 (3-4) |  | 4 (3-4) | 4 (2-4) |  | 3 (2-3) | 4 (2-4) |  | 4 (2-4) | 3.5 (3-4) |  |
| **PAM-13 – hospital admission** item12:  mean±SD | 3.23±0.6 | | 3.05±0.8 | 0.68 | 3.4±0.89 | 3.33±0.5 | 0.9 | 3.2±0.44 | 3.3±0.51 | 0.84 | 2.6±0.57 | 3.3±0.57 | 0.28 | 3.6±0.55 | 3±0.81 | 0.28 |
| Median (min-max) | 3 (3-5) | | 3 (1-4) |  | 3 (3-5) | 3 (3-4) |  | 3 (3-4) | 3 (3-4) |  | 3 (2-3) | 3 (3-4) |  | 4 (3-4) | 3 (2-4) |  |
| **PAM-13 – hospital admission** item13:  mean±SD | 3.2±0.78 | | 3.77±0.55 | 0.05* | 3.4±0.89 | 3.66±0.8 | 0.84 | 3.4±0.89 | 4±0 | 0.33 | 3±0 | 4±0 | 0.00 | 3.6±0.89 | 4±04 (4-4) | 0.5 |
| Median (min-max) | 3 (2-4) | | 4 (3-5) |  | 4 (2-4) | 3.5 (3-4) |  | 4 (2-4) | 4 (4-4) |  | 3 (3-3) | 4 (4-4) |  | 4 (2-4) |  |  |
| **PAM-13 – POD14**  total raw:  mean±SD | 42.57±7.7 | 45.2±5.1 | | 0.22 | 45±6.92 | 43.1±7.0 | 0.67 | 47.4±4.2 | 48.6±3.5 | 0.68 | 33.6±8.0 | 48.6±3.5 | 0.07 | 43.2±2.2 | 46.5±4.79 | 0.31 |
| Median (min-max) | 43 (24-56) | 45 (33-52) | |  | 42 (38-56) | 45.5 (33-50) |  | 48 (41-52) | 49 (45-52) |  | 29 (29-43) | 49 (45-52) |  | 43 (41-47) | 46 (42-52) |  |
| **PAM-13 – POD14**  Item 1:  mean±SD | 3.3±0.73 | | 3.66±0.59 | 0.12 | 3±1 | 3.3±0.81 |  | 3.6±0.54 | 4±0 |  | 3.3±1.15 | 4±0 |  | 3.4±0.54 | 4±0 |  |
| Median (min-max) | 3 (2-4) | | 4 (2-4) |  | 3 (2-4) | 3.5 (2-4) | 0.55 | 4 (3-4) | 4 (4-4) | 0.32 | 4 (2-4) | 4 (4-4) | 0.41 | 3 (3-4) | 4 (0-4) | 0.1 |
| **PAM-13 – POD14**  Item 2:  mean±SD | 3.6±0.49 | | 3.7±0.54 | 0.19 | 3.4±0.54 | 3.5±0.83 | 0.68 | 3.8±0.44 | 4±0 | 0.6 | 3.8±0.44 | 4 ±0 | 0.51 | 3.6±0.57 | 4±0 | 0.23 |
| Median (min-max) | 4 (3-4) | | 4 (2-4) |  | 3 (3-4) | 4 (2-4) |  | 4 (3-4) | 4 (4-4) |  | 4 (3-4) | 4 (4-4) |  | 4 (3-4) | 4 (4-4) |  |
| **PAM-13 – POD14**  Item 3:  mean±SD | 3.3±0.67 | | 3.55±0.61 | 0.4 | 3.8±0.44 | 3.5±0.83 | 0.64 | 3.6±0.54 | 3.6±0.57 | 1 | 3±1 | 3.6±0.57 | 0.45 | 3.4±0.54 | 3.75±0.5 | 0.39 |
| Median (min-max) | 3 (2-4) | | 4 (2-4) |  | 4 (3-4) | 4 (2-4) |  | 4 (3-4) | 4 (3-4) |  | 3 (2-4) | 4 (3-4) |  | 3 (3-4) | 4 (3-4) |  |
| **PAM-13 – POD14**  Item 4:  mean±SD | 3.3±0.85 | | 3.55±0.7 | 0.34 | 3.4±1.14 | 3.3±0.81 | 0.81 | 3.8±0.45 | 4±0 | 0.6 | 2.6±1.15 | 4±0 | 0.18 | 3.4±0.54 | 3.25±0.95 | 1 |
| Median (min-max) | 3 (2-5) | | 4 (2-4) |  | 3 (2-5) | 3.5 (2-4) |  | 4 (3-4) | 4 (4-4) |  | 2 (2-4) | 4 (4-4) |  | 3 (3-4) | 3.5 (2-4) |  |
| **PAM-13 – POD14**  Item 5:  mean±SD | 3.4±0.68 | | 3.5±0.62 | 1 | 3.6±0.54 | 3±0.44 | 0.15 | 3.8±0.45 | 3.6±0.57 | 0.84 | 3.6±0.57 | 3.6±0.57 | 1.00 | 3±0.7 | 3.75±0.5 | 0.14 |
| Median (min-max) | 4 (2-4) | | 4 (2-4) |  | 4 (3-4) | 4 (3-4) |  | 4 (3-4) | 4 (3-4) |  | 4 (3-4) | 4 (3-4) |  | 3 (2-4) | 4 (3-4) |  |
| **PAM-13 – POD14**  Item 6:  mean±SD | 3.3±0.8 | | 3.66±0.48 | 0.29 | 3.4±0.54 | 3.5±0.54 | 0..83 | 3.6±0.54 | 4±0 | 0.32 | 3.6±0.57 | 4±0 | 0.36 | 3.2±0.83 | 3.5±0.57 | 0.68 |
| Median (min-max) | 4 (1-4) | | 4 (3-4) |  | 3 (3-4) | 3.5 (3-4) |  | 4 (3-4) | 4 (4-4) |  | 4 (3-4) | 4 (4-4) |  | 3 (2-4) | 3.5 (3-4) |  |
| **PAM-13 – POD14**  Item 7:  mean±SD | 3.5±0.75 | | 3.77±0.43 | 0.34 | 3.6±0.89 | 3.5±0.54 | 0.59 | 3.8±0.44 | 4±0 | 0.6 | 3.3±1.15 | 4±0 | 0.41 | 3.4±0.55 | 4±0 | 0.1 |
| Median (min-max) | 4 (2-4) | | 4 (3-4) |  | 4 (2-4) | 3.5 (3-4) |  | 4 (3-4) | 4 (4-4) |  | 4 (2-4) | 4 (4-4) |  | 3 (3-4) | 4 (4-4) |  |
| **PAM-13 – POD14**  Item 8:  mean±SD | 3.2±0.95 | | 3.55±0.78 | 0.38 | 4±0.7 | 3.5±1.22 | 0.44 | 3.8±0.44 | 4±0 | 0.6 | 2±1 | 4±0 | 0.07 | 3±0.7 | 3.5±0.57 | 0.33 |
| Median (min-max) | 3 (1-5) | | 4 (2-8) |  | 4 (3-5) | 4 (2-5) |  | 4 (3-4) | 4 (4-4) |  | 2 (1-3) | 4 (4-4) |  | 3 (2-4) | 3.5 (3-4) |  |
| **PAM-13 – POD14**  Item 9:  mean±SD | 3.1±1.15 | | 3.2±0.64 | 0.7 | 3.6±1.14 | 3.1±0.75 | 0.46 | 3.8±0.44 | 3.6±0.57 | 0.84 | 1±0 | 3.6±0.57 | 0.09 | 3.2±0.83 | 3.25±0.5 | 1 |
| Median (min-max) | 3.5 (1-5) | | 3 (2-4) |  | 4 (2-5) | 3 (2-4) |  | 4 (3-4) | 4 (3-4) |  | 1 (1-1) | 4 (3-4) |  | 3 (2-4) | 3 (3-4) |  |
| **PAM-13 – POD14**  Item 10: |  | |  |  |  |  |  |  |  |  |  |  |  |  |  |  |
| mean±SD | 3.2±0.95 | | 3.16±0.7 | 0.58 | 3.6±0.89 | 3.33±0.5 | 0.35 | 3.4±0.89 | 3.3±0.57 | 0.87 | 2.5±0.7 | 3.3±0.57 | 0.25 | 3.6±0.54 | 3.25±0.5 | 0.39 |
| Median (min-max) | 3.5 (1-4) | | 3 (1-4) |  | 4 (2-4) | 3 (3-4) |  | 4 (2-4) | 3 (3-4) |  | 2.5 (2-3) | 3 (3-4) |  | 4 (3-4) | 3 (3-4) |  |
| **PAM-13 – POD14**  Item 11:  mean±SD | 3.14±0.9 | | 3.27±0.67 | 0.63 | 3.6±0.89 | 3.1±0.75 | 0.54 | 3.4±0.54 | 3.3 ±0.57 | 1 | 2.3±0.57 | 3.3±0.57 | 0.15 | 3.2±0.83 | 3.25±0.95 | 0.93 |
| Median (min-max) | 3 (1-5) | | 3 (2-4) |  | 3 (3-5) | 3 (2-4) |  | 3 (3-4) | 3 (3-4) |  | 2 (2-3) | 3 (3-4) |  | 3 (2-4) | 3.5 (2-4) |  |
| **PAM-13 – POD14**  Item 12:  mean±SD | 3.31±0.82 | | 3.22±0.64 | 0.61 | 3.2±1.3 | 3±0.63 | 0.74 | 3.2±0.44 | 3.66±0.57 | 0.29 | 4±NaN | 3.66±0.57 | 0.55 | 3.6±0.54 | 3.5±0.57 | 0.88 |
| Median (min-max) | 3(2-5) | | 3(2-4) |  | 3(2-5) | 3(2-4) |  | 3(3-4) | 4(3-4) |  | 4(4-4) | 4(3-4) |  | 4(3-4) | 3.5(3-4) |  |
| **PAM-13 – POD14**  Item 13:  mean±SD | 3.04±0.8 | | 3.27±0.67 | 0.38 | 2.8±0.83 | 3.33±0.81 | 0.31 | 3.8±0.44 | 3.33±0.57 | 0.29 | 2.33±0.57 | 3.33±0.57 | 0.15 | 3.2±0.44 | 3.5±0.57 | 0.45 |
| Median (min-max) | 3(2-4) | | 3(2-4) |  | 3(2-4) | 3.5 (2-4) |  | 4(3-4) | 3 (3-4) |  | 2(2-3) | 3(3-4) |  | 3(3-4) | 3.5 (3-4) |  |
| **EQ-5D – hospital admission** utility score  mean±SD | 7.1±1.81 | | 7.11±2.99 | 0.76 | 5.6±0.89 | 6.33±1.5 | 0.48 | 8.4±1.14 | 10.6±3.0 | 0.17 | 8±3 | 10.6±3.0 | 0.43 | 6.2±0.44 | 6.5±1 | 0.86 |
| Median (min-max) | 7 (5-11) | | 6.5 (5-14) |  | 5 (5-7) | 6 (5-8) |  | 8 (7-10) | 10 (8-14) |  | 8 (5-11) | 10 (8-14) |  | 6 (6-7) | 6 (6-8) |  |
| **EQ-5D – hospital admission** VAS  mean±SD | 70.7±16 | | 71.1±21.11 | 0.73 | 80±10 | 70±33.4 | 0.73 | 71±11.4 | 63.3±15 | 0.44 | 53.3±15 | 63±15.2 | 0.37 | 77±15.6 | 70±8.1670 (60- | 0.44 |
| Median (min-max) | 70 (35-100) | | 75 (10-100) |  | 80 (70-90) | 90 (10-100) |  | 70 (60-90) | 60 (50-80) |  | 50 (40-70) | 60 (50-80) |  | 80 (60-100) | 80) |  |
| **EQ-5D – hospital discharge** utility score  mean±SD | 8.7±2.19 | | 8.94±2.9 | 0.84 | 9±2.55 | 9.5±2.66 | 0.76 | 9.6±1.95 | 11±2.64 | 0.42 | 10.3±1.5 | 11±2.64 | 0.78 | 6.4±1.14 | 6±0.81 | 0.57 |
| Median (min-max) | 9 (5-13) | | 9 (5-13) |  | 9 (6-13) | 9.5 (5-13) |  | 9 (7-12) | 12 (8-13) |  | 10 (9-12) | 12 (8-13) |  | 6 (5-8) | 6 (5-7) |  |
| **EQ-5D – hospital discharge** VAS  mean±SD | 56±21.9 | | 51±16.72 | 0.51 | 51±27 | 44±20.5 | 0.64 | 65±3.78 | 53±25.1 | 0.3 | 40±10 | 53±25.1 | 0.14 | 56±33.0 | 62.5±9.57 | 0.72 |
| Median (min-max) | 60 (5-9) | | 50 (20-80) |  | 50 (20-82) | 47.5 (20-70) |  | 65 (60-70) | 50 (30-80) |  | 40 (30-50) | 50 (30-80) |  | 60 (5-95) | 65 (50-70) |  |
| **EQ-5D – POD14** Utility score  mean±SD | 8.75±2.1 | | 8.94±2.9 | 0.84 | 9±2.55 | 9.5±2.66 | 0.76 | 9.6±1.95 | 11±2.64 | 0.42 | 10.3±1.5 | 11±2.64 | 0.78 | 6.4±1.14 | 6±0.81 | 0.57 |
| Median (min-max) | 9 (5-13) | | 9 (5-13) |  | 9 (6-13) | 9.5 (5-13) |  | 9 (7-12) | 12 (8-13) |  | 10 (9-12) | 12 (8-13) |  | 6 (5-8) | 6 (5-7) |  |
| **EQ-5D – POD14** VAS  mean±SD | 62±21.1 | | 60.4±31.31 | 0.9 | 0±15.81 | 55±33.5 | 0.39 | 55±5.77 | 40±26.4 | 0.24 | 50±10 | 40±26.4 | 0.68 | 69±37.9 | 95±5.77 | 0.36 |
| Median (min-max) | 60 (7-100) | | 60 (7-100) |  | 70 (50-90) | 65 (14-90) |  | 55 (50-60) | 50 (10-60) |  | 50 (40-60) | 50 (10-60) |  | 70 (7-100) | 95 (90-100) |  |
| **PANAS – POSITIVE - hospital admission**  mean±SD | 28.6±7.7 | | 27.88±8.43 | 0.82 | 28±8.51 | 26±11.5 | 0.79 | 34±10.3 | 31±9.89 | 0.82 | 27±1 | 31±9.89 | 0.66 | 29.5±5.0 | 31±4.54 | 0.63 |
| Median (min-max) | 26.5 (17-50) | | 28 (7-39) |  | 26 (17-38) | 28 (7-39) |  | 31 (25-50) | 31 (24-38) |  | 27 (26-28) | 31 (24-38) |  | 27.5 (26-37) | 30 (27-37) |  |
| **PANAS – NEGATIVE - hospital admission**  mean±SD | 21.9±8.4 | | 18.05±6.68 | 0.12 | 21±12.7 | 15.8±5.9 | 0.33 | 18±6.36 | 16.5±3.5 | 0.12 | 25.6±6.6 | 16.5±3.5 | 0.12 | 22.7±11 | 19±7.39 | 0.54 |
| Median (min-max) | 22.5 (10-38) | | 17 (10-32) |  | 19 (10-38) | 15 (10-26) |  | 19 (10-24) | 16.5 (14-19) |  | 29 (18-30) | 16.5 (14-19) |  | 23.5 (10-34) | 20 (10-26) |  |
| **PANAS – POSITIVE - hospital discharge**  mean±SD | 26.5±7.8 | | 27.27±8.26 | 0.74 | 20.6±6.6 | 21.8±5.5 | 0.74 | 34.6±8.1 | 39.6±1.5 | 0.34 | 30±1.73 | 39.6±1.5 | 0.01 | 26.8±2.5 | 28.28±6.5 | 0.38 |
| Median (min-max) | 28 (12-46) | | 27 (14-41) |  | 23 (12-29) | 23.5 (15-27) |  | 34 (24-46) | 40 (38-41) |  | 31 (28-31) | 40 (38-41) |  | 28 (24-29) | 30 (19-34) |  |
| **PANAS – NEGATIVE - hospital discharge**  mean±SD | 17.4±9.0 | | 17.05±6.99 | 0.84 | 19.8±10 | 18.6±8.9 | 0.84 | 13.2±3.9 | 12.6±3.7 | 0.85 | 13.6±4.7 | 12.6±3.7 | 0.81 | 14.8±7.5 | 18.5±7.68 | 0.53 |
| Median (min-max) | 13 (10-43) | | 15 (10-31) |  | 19 (10-36) | 17 (10-31) |  | 11 (10-18) | 11 (10-17) |  | 12 (10-19) | 11 (10-17) |  | 13 (10-28) | 19.5 (10-28) |  |
| **PANAS – POSITIVE – POD14**  mean±SD | 27.2±7.8 | | 29.3±8.56 | 0.41 | 26±7.21 | 26±12.0 | 1 | 35.2±3.8 | 37±4.35 | 0.39 | 25.6±13 | 37±4.35 | 0.26 | 27.4±5.4 | 29.25±6.94 | 0.66 |
| Median (min-max) | 28.63 (13-39) | | 30.5 (12-43) |  | 24 (16-34) | 25.5 (12-43) |  | 35.25 (29-39) | 39 (32-42) |  | 25 (13-39) | 39 (32-40) |  | 30 (21-32) | 30.5 (21-35) |  |
| **PANAS – NEGATIVE – POD14**  mean±SD | 14.7±5.1 | | 14.16±5.06 | 0.49 | 15±5.96 | 14.5±5.2 | 1 | 11.2±1.0 | 12.6±4.6 | 0.76 | 14.6±2.3 | 12.6±4.6 | 0.65 | 15.6±8.2 | 17±7.7 | 0.9 |
| Median (min-max) | 13 (10-330) | | 12 (10-27) |  | 11 (10-22) | 12.5 (10-22) |  | 11 (10-13) | 10 (10-18) |  | 16 (12-16) | 10 (10-18) |  | 12 (10-30) | 15.5 (10-27) |  |
| **SWEMWB – POD 14 (raw score)**  mean±SD | 25.4±5.3 | | 25.7±3.28 | 0.83 | 24.2±2.3 | 24.5±4.5 | 0.89 | 30±3.16 | 27.6±0.5 | 0.28 | 24.6±8.0 | 27.6±0.5 | 0.58 | 27.2±3.1 | 27±2.58 | 0.92 |
| Median (min-max) | 26 (13-35) | | 26.5 (19-30) |  | 24 (21-27) | 24 (19-30) |  | 29 (27-35) | 28 (27-28) |  | 26 (16-32) | 28 (27-28) |  | 27 (24-32) | 27 (24-32) |  |
| **SWEMWB – POD 14 (metric score)**  mean±SD | 23.3±4.6 | | 23.02±2.76 | 0.88 | 21.7±1.3 | 22.2±3.7 | 0.8 | 27.6±4.3 | 24.7±0.5 | 0.28 | 22.9±6.4 | 24.7±0.5 | 0.68 | 24.47±3 | 24.2±2.36 | 0.89 |
| Median (min-max) | 23.21 (14-35) | | 23.6 (17-27) |  | 21.5 (19-24) | 21.5 (17-27) |  | 26.02 (24-35) | 25.03 (24-25) |  | 23.21 (16-29) | 25.03 (24-25) |  | 24.11 (21-29) | 24.12 (21-27) |  |
| **GSE - hospital admission**  mean±SD | 30.0±5.7 | | 31.8±4.56 | 0.3 | 28.4±6.2 | 31.3±4.8 | 0.4 | 31.8±6.0 | 36.6±1.5 | 0.23 | 26.3±4.0 | 36.6±1.5 | 0.04 | 34.2±3.7 | 32.2±3.94 | 0.47 |
| Median (min-max) | 30 (20-40) | | 31 (24-39) |  | 27 (20-36) | 30.5 (27-39) |  | 30 (24-40) | 37 (35-38) |  | 27 (22-30) | 37 (35-38) |  | 34 (30-40) | 31 (29-38) |  |
| **GSE – POD14**  mean±SD | 30±6.85 | | 30.94±4.34 | 0.86 | 28±5.29 | 29.5±5.5 | 0.66 | 34.6±4.2 | 31.3±2.3 | 0.37 | 25.6±9.0 | 31.3±2.3 | 0.39 | 33.2±5.3 | 33.25±1.7 | 0.98 |
| Median (min-max) | 32 (17-40) | | 30.5 (24-39) |  | 30 (20-34) | 27.5 (24-39) |  | 35 (29-40) | 30 (30-34) |  | 25 (17-35) | 30 (30-34) |  | 34 (25-40) | 33.5 (31-35) |  |
| **Communication** (POD14) Physician  communication general  mean±SD  Median (min-max)  availability  mean±SD  Median (min-max)  content  mean±SD  Median (min-max) | 8.5±1.56  9.0 (5-10)  8.9±1.28  9.0 (5-10)  8.6±1.56  9.0 (5-10) | | 8.24±2.02  9.0 (2-10)  8.18±2.04  9.0 (2-10)  7.76±2.51  8.5 (1-10) | 0.69  0.25  0.29 | 8.6±1.5  9.0 (6-10)  9.6±0.52  10.0 (9.0-10)  9.0±1.55  10.0 (7-10) | 7.8±2.97  9.0 (2-10)  7.71±2.7  10.0 (2-10)  7.5±2.76  10.0 (2-10) | 0.94  0.08  0.2 | 8.6±1.85  9.0 (5-10)  8.8±1.94  9.5 (5-10)  8.8±1.94  9.5 (5-10) | 8.25±1.7  8.5 (6-10)  8.75±1.8  9.5 (6-10)  7.0±4.08  8.5 (1-10) | 0.74  0.99  0.44 | 9.67±0.5  10.0 (9-10)  8.67±1.1  8.0 (8-10)  9.0±1.73  10.0 (7-10.0) | 9.5±0.71  9.5 (9-10)  8.0±2.83  8.0 (6-10)  9.0±1.41  9.0 (8-10.0) | 0.80  0.79  1.00 | 7.83±1.6  8.0 (6-10)  8.5±1.05  8.5 (7-10)  7.83±1.1  8.0 (6-9) | 8.25±0.83  8.25 (7-9)  8.5±0.87  9.0 (7-9)  8.25±0.83  8.25 (7-9.0) | 0.61  1  0.52 |
| **Communication** (POD14)Nurses  communication general  mean±SD  Median (min-max)  availability  mean±SD  Median (min-max)  content  mean±SD  Median (min-max) | 9.05±1.2  9.05 (6-10)  9.0±1.41  9.0 (5-10)  9.07±1.1  9.07 (5-10) | | 8.41±2.03  9.0 (2-10)  8.06±1.8  8.03 (3-10)  8.0±2.28  8.0 (1-11) | 0.33  0.04  NaN | 8.8±1.47  9.5 (7-10)  9.6±0.52  10.0 (9-10)  9.33±0.8  9.5 (8-10) | 7.86±2.9  9.5 (2-10)  7.57±2.3  10.0 (3-10)  8.4±2.21  9.5 (5-11) | 0.7  0.03  NaN | 9.8±0.4   10.0 (9-10)  8.8±1.94  9.5 (5-10)  8.8±1.94  9.5 (5-10) | 9.0±1.41  9.5 (7-10)  8.5±1.91  9.0 (6-10)  7.0±4.08  8.5 (1-10) | 0.47  0.9  NaN | 9.67±0.5  10.0 (9-10)  9.33±1.1  10.0 (8-10)  9.0±1.0  9.0 (8-10) | 10.0±0.0  10.0 (10-10)  8.5±2.12  8.5 (7-10)  9.0±1.41  9.0 (8-10) | 0.42  0.68  1.00 | 8.33±1.3  8.5 (6-10)  8.33±1.5  9.0 (6-10) | 8.0±0.71  8.0 (7-9)  8.25±0.83  8.25 (7-9) | 0.63  0.91 |
| **Caregivers** | | | | | | | | | | | | | | | | |
| **VAS stress – hosp admission**  Mean±SD | 52±18.9 | | 77.78±15.6 |  | 43±15.9 | 66±17.9 |  |  | 100±0 |  | 83±15.2 | 100±0 | 0.19 | 20±6.32 |  |  |
| Median (min-max) | 52.5 (10-100) | | 77.78 (40-100.0 ) |  | 43.33 (20-70) | 66.67 (40-90) |  |  | 100.0 (100 -100) |  | 80.0 (70-100) | 100.0 (100-100) |  | 20.0 (10-30) |  |  |
| **VAS stress – hosp discharge**  Mean±SD | 31±32.3 | | 41.1±16.9 | 0.015* | 33±49.3 | 43.3±15 | 0.54 |  | 50±NaN |  | 30±26.4 | 50±NaN | 0.16 | 30±NaN |  |  |
| Median (min-max) | 30 (0-90) | | 50 (10-60) |  | 10 (0-90) | 50 (20-60) |  |  | 50 (50-50) |  | 40 (0-50) | 50 (50-50) |  | 30 (30-30) |  |  |
| **VAS stress – POD14**  Mean±SD | 15±10.4 | | 1.7±18.16 |  | 16±18.2 | 25±29.0 |  |  |  |  | 13±15.2 | 0.0±0.0 | 0.15 | 20±0 |  |  |
| Median (min-max) | 15.71 (0-50) | | 21.71 (0-80) |  | 16.67 (0-50) | 25.33 (0-80) |  |  |  |  | 10.0 (0-30) | 0.0 (0-0) |  | 20 (20-20) |  |  |
| **SWEMWB – POD14 (Raw Score)**  Mean±SD | 32.0±1.1 | | 23.71±4.52 |  | 32±1.59 | 23.3±7.5 |  |  |  |  | 31.6±2.5 | 26.0±0.0 | 0.05 | 31.0±0.0 |  |  |
| Median (min-max) | 32.0 (29-35) | | 23.71 (9-31) |  | 32.67 (30-35) | 23.33 (9-31) |  |  |  |  | 32.0 (29-34) | 26.0 (26-26) |  | 31.0 (31-31) |  |  |
| **SWEMWB – POD14 (Metric Score)**  Mean±SD | 29.6±1.7 | | 21.89±3.45 | NaN | 31.0±2.5 | 21.6±5.7 | NaN |  |  |  | 29.2±3.2 | 23.2±0.0 | 0.16 | 28.1±0.0 |  |  |
| Median (min-max) | 29.67 (26-35) | | 21.89 (11- 28) |  | 31.02 (27-35) | 21.66 (11-28) |  |  |  |  | 29.31 (26-32) | 23.21 (23-23) |  | 28.13 (28-28) |  |  |
| **GSE –POD14**  Mean±SD | 32.8±3.7 | | 26.3±12.09 | 0.000001* | 33.6±4.7 | 26.3±12 | 0.22 |  |  |  | 31.3±3.5 | NaN±NN | NaN |  |  |  |
| Median (min-max) | 32 (28-39) | | 30 (3-38) | ** | 32 (30-39) | 30 (3-38) |  |  |  |  | 31 (28-35) | NaN (-) |  |  |  |  |

POD: PostOperative Day

# NaN = Not Available
