# Supplementary figures and images for: Stress Reduction in Perioperative Care: Feasibility Randomized Controlled Trial
Source: J Med Internet Res. 2025 Jan 7;27:e54049. doi: 10.2196/54049 (PMC11751654; doi:10.2196/54049)

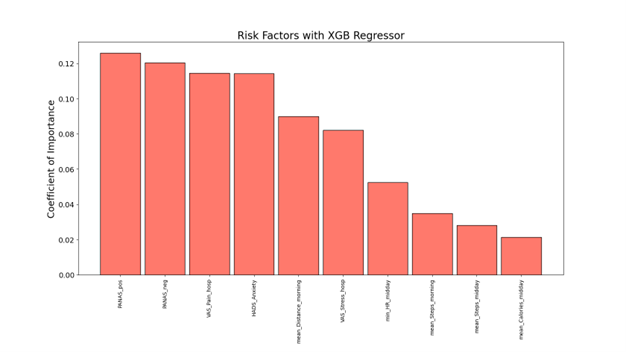

Supplement: Multimedia Appendix 5 [file jmir_v27i1e54049_app5.png]

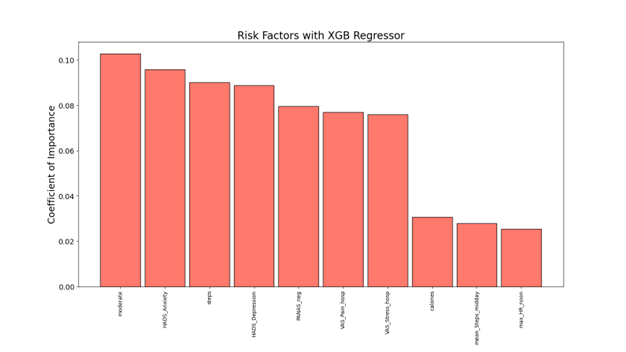

Supplement: Multimedia Appendix 6 [file jmir_v27i1e54049_app6.png]

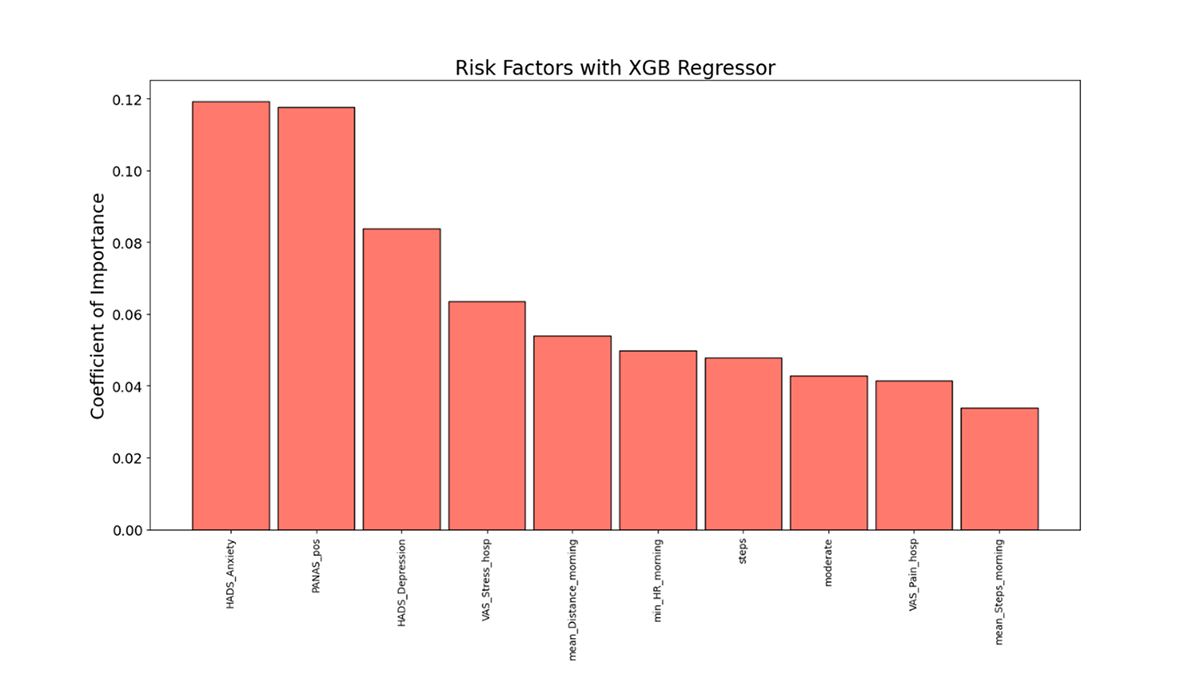

Supplement: Multimedia Appendix 7 [file jmir_v27i1e54049_app7.png]

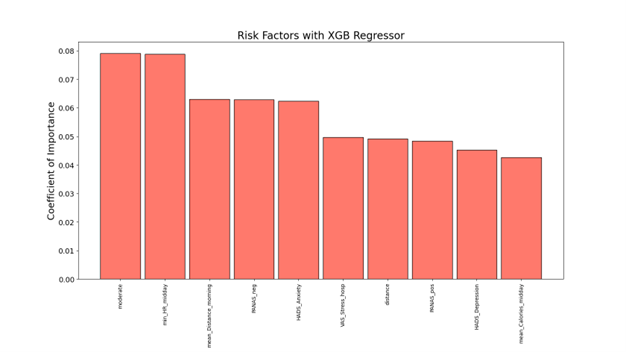

Supplement: Multimedia Appendix 8 [file jmir_v27i1e54049_app8.png]

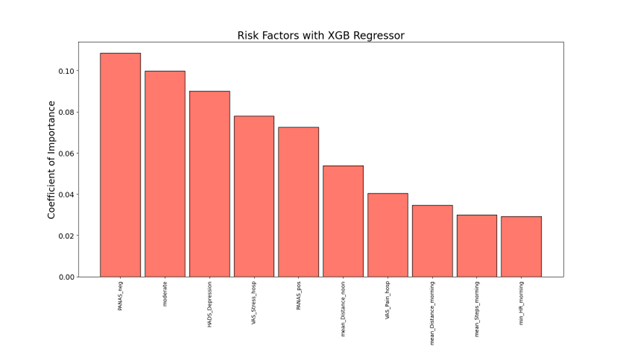

Supplement: Multimedia Appendix 9 [file jmir_v27i1e54049_app9.png]

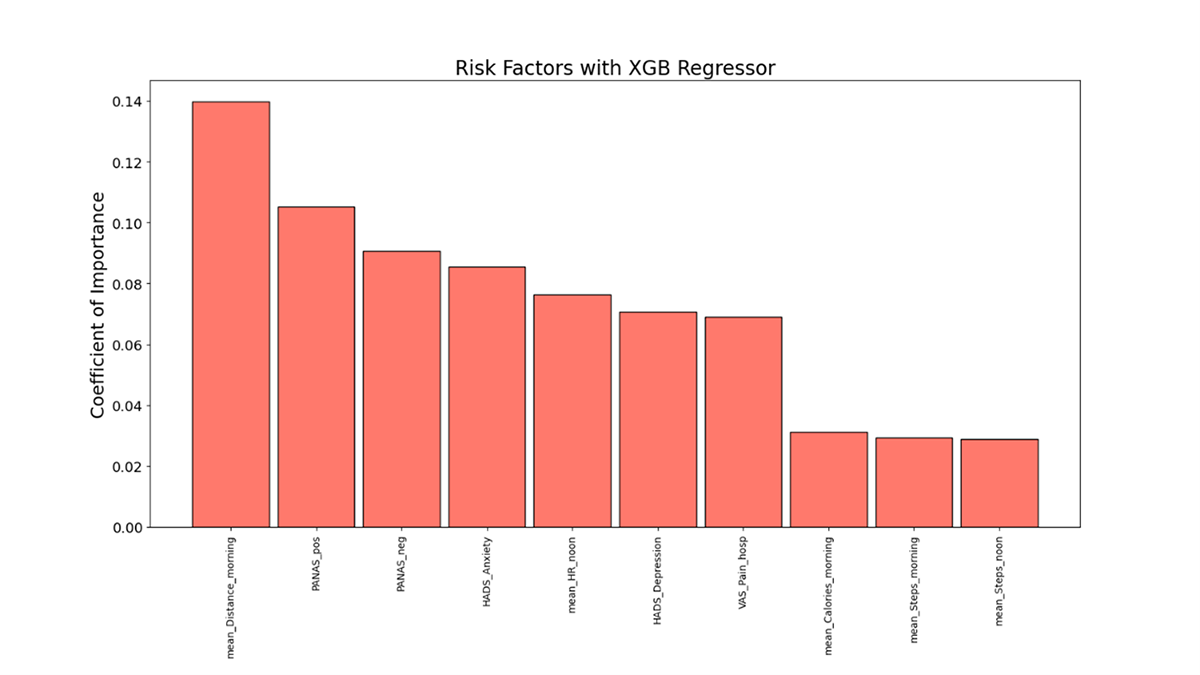

Supplement: Multimedia Appendix 10 [file jmir_v27i1e54049_app10.png]
